# Numerical simulation of the layered filling process of cemented paste backfill based on thermo-hydro-mechanical-chemical coupling analysis

**DOI:** 10.1038/s41598-026-51983-0

**Published:** 2026-05-09

**Authors:** Shifei Yang, Zongyong Wang, Kepeng Hou, Yalei Zhe, Qunzhi Cheng, Yanlin Li

**Affiliations:** 1 Faculty of Land Resources Engineering, Kunming University of Science and Technology, Kunming, 650093 China; 2 Yunnan Phosphate Chemical Group Co., Ltd, Kunming, 650600 China; 3 Key Laboratory of Development and Utilization of Blue Mines, Special Underground Space in Yunnan Province, Kunming, 650093 China

**Keywords:** CPB, THMC coupling, Layered backfilling, Spatiotemporal evolution, Numerical simulation, Energy science and technology, Engineering, Materials science

## Abstract

Layered backfilling is a critical construction method for ensuring the overall stability of backfill in deep mines. Its cyclic “fill-cure-refill” operation mode induces complex thermo-hydro-mechanical-chemical (THMC) coupling effects and significant spatiotemporal heterogeneity within the backfill body. Addressing the limitation that existing research predominantly focuses on single continuous filling and lacks in-depth investigation into the physical field transfer mechanisms at layered interfaces, this paper establishes a fully coupled THMC numerical simulation model for Cemented Paste Backfill (CPB) considering a time-varying computational domain. On this basis, the influence laws of the cement-sand ratio (c/s ratio), inter-layer interval time, and layering strategy (continuous, two-layer, and three-layer) on the spatiotemporal evolution of temperature, seepage, and stress fields were systematically analyzed. Results indicate that the c/s ratio is the primary driver of multi-field evolution; higher ratios increase peak temperatures and matrix suction rates, enhancing early strength. Interval time governs pore water pressure (PWP) dissipation; longer interval utilizes a “peak-shifting effect” to reduce heat accumulation and improve vertical stress. Furthermore, a three-layer strategy creates “sawtooth-like” PWP dissipation, effectively preventing the high-pressure accumulation and stress lag associated with continuous filling. This work clarifies THMC mechanisms at layered interfaces, providing a theoretical basis for optimizing backfill consolidation.

## Introduction

The continuous exploitation of mineral resources provides a vital material foundation for global economic development, but it also brings severe environmental challenges and safety hazards. Traditional surface tailings storage facilities not only occupy vast amounts of land resources but also face high-risk issues such as dam failures, acid mine drainage leakage, and dust pollution, which have become bottlenecks constraining the sustainable development of the mining industry^1–3^. With the increasingly strict environmental regulations worldwide and the popularization of the “Green Mine” concept, seeking an efficient, safe, and environmentally friendly method for tailings disposal has become an industry consensus. Cemented Paste Backfill (CPB) technology has emerged and been widely adopted in this context^4–6^. This technology involves mixing full tailings, water, and binders in specific proportions to prepare a high-density, non-segregating slurry, which is then pumped into underground stopes. CPB technology not only effectively controls ground pressure activities and prevents surface subsidence but also maximizes the underground disposal of tailings, thereby resolving the safety hazards of tailings dams at the source and offering significant economic and social benefits^7–9^.

**Fig. 1:**
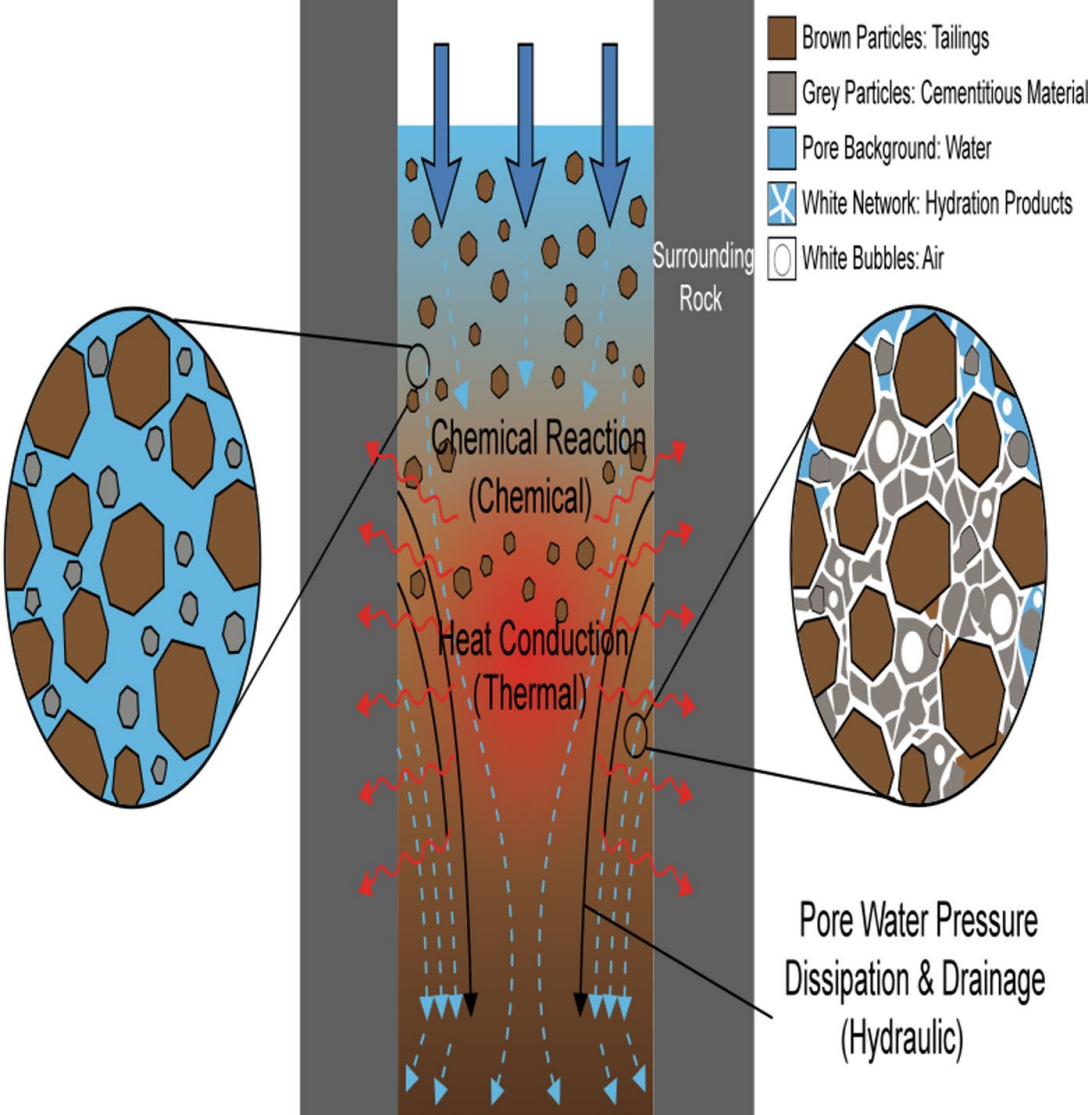
Schematic representation of the multi-physics coupling mechanisms and micro-structural evolution within cemented paste backfill.

Once the backfill slurry is transported to the stope, its physical and mechanical state undergoes drastic changes, transforming from an initial fluid-like suspension into a porous solid medium with measurable strength^10^. As schematically illustrated in Fig. 1, this process is not a simple physical sedimentation but a complex evolutionary system involving strong multi-physics coupling. Specifically, the hydration reaction of binders (such as cement) consumes pore water (chemical-hydraulic coupling), leading to a reduction in pore water pressure (PWP) and the generation of matric suction. Simultaneously, the generation of hydration products fills the pore spaces, significantly reducing the porosity and permeability of the material (chemical-mechanical coupling), which in turn hinders the further drainage of pore water^11–16^. Furthermore, as the overlying slurry accumulates, the lower backfill body undergoes consolidation deformation under self-weight stress, and the compression of the skeleton conversely affects the dissipation rate of pore water pressure (mechanical-hydraulic coupling)^17–19^. Therefore, accurately revealing this thermo-hydro-mechanical-chemical (THMC) coupling mechanism is essential for predicting the early-age strength development, pore water pressure distribution, and final consolidation settlement of the backfill.

In practical engineering applications, to ensure the safety of backfilling operations, particularly to prevent the failure of backfill barricades due to excessive instantaneous hydrostatic pressure, layered backfilling has become a standard industrial practice^20^. Unlike idealized continuous backfilling, layered backfilling employs a cyclic “fill-rest-refill” operational mode. This intermittent construction process makes the consolidation process of the backfill extremely complex: during the resting period, the lower backfill layer undergoes partial hydration and consolidation, potentially forming a “crust” with low permeability on its surface. When the new upper slurry is placed, the lower old backfill layer acts as both a load-bearing body and a potential drainage channel^12,14^. This interlayer interaction causes the dissipation path of pore water pressure to be discontinuous, and the transfer of total stress is jointly influenced by the “arching effect” and the interlayer interface^15,16^. If the resting time is too short, the insufficient strength of the lower backfill may lead to overall instability; if the resting time is too long, it will affect the mining cycle and reduce production efficiency.

Although layered backfilling is prevalent in mines, existing research efforts still have certain limitations. In terms of experimental research, most laboratory tests focus on the effects of curing age and mix proportions on the mechanical properties of hardened backfill, making it difficult to replicate the continuous loading process of on-site layered casting^21–25^. In terms of theoretical analysis, classical Terzaghi consolidation theory or Gibson’s large strain theory typically assume constant material parameters or fixed boundary conditions, which are difficult to apply to complex media like CPB where material properties change in real-time due to hydration^26^. Regarding numerical simulation, although some scholars have established models considering HMC coupling, most models, when dealing with layered backfilling, simplify it as instantaneous loading or pre-set static layers, ignoring the Moving Boundary problem during the dynamic rising of the slurry surface^27–30^. This simplification overlooks the process where the self-weight of the slurry is gradually applied, often leading to distorted predictions of early-age pore water pressure peaks. Moreover, quantitative research on how the resting time, filling rate, and cement-to-tailings ratio synergistically affect the multi-physics evolution within the backfill remains scarce.

In light of this, this paper aims to establish a fully coupled HMC numerical analysis model for CPB that can accurately describe the entire process of layered backfilling. This paper introduces dynamic mesh technology based on the Arbitrary Lagrangian-Eulerian (ALE) method to achieve real-time tracking of the rising slurry surface and intermittent pauses, thereby overcoming the deficiencies of traditional models in dealing with time-dependent computational domains. On this basis, this paper systematically conducts a parametric study to deeply explore the effects of the cement-to-tailings ratio, interlayer resting time, and layering strategies on the dissipation of pore water pressure, the development of vertical effective stress, and consolidation settlement characteristics within the backfill. The results aim to reveal the multi-physics transfer mechanisms under layered filling conditions, providing a solid theoretical basis for optimizing mining backfill parameters, scientifically setting resting times, and ensuring barricade safety.

### Numerical model of THMC multi-field coupling in backfill

With the deepening understanding of the coupling mechanisms in backfill, significant progress has been made in the mathematical modeling of multi-field coupling effects, strongly advancing work in this field.

**Fig. 2:**
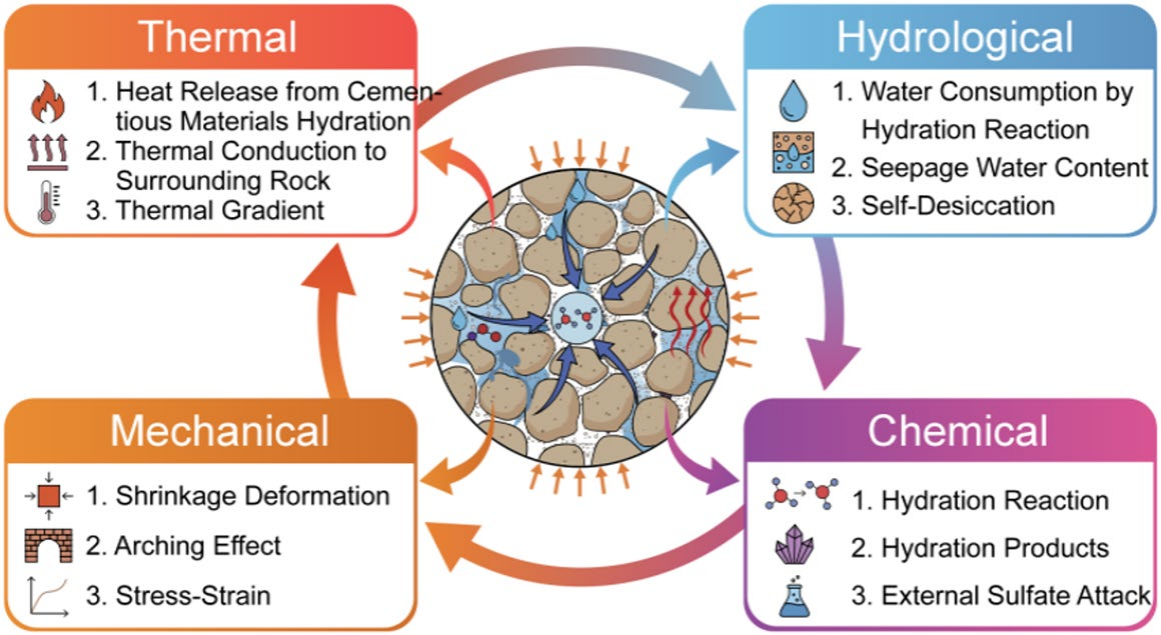
THMC multi-field coupling Interactions in Backfill.

Figure 2 presents the multi-physical processes involved in backfill, including thermal response, hydraulic behavior, mechanical performance, and chemical activity. These processes are interlinked and exhibit strong couplings, which collectively drive the evolution of backfill strength. Considerable research efforts have led to the development of both individual mathematical descriptions for each process and integrated Thermo‑Hydro‑Mechanical‑Chemical (THMC) coupled frameworks [11‑19,27‑30].

### Chemical process

The chemical evolution of backfill primarily involves the hydration of its cementitious components. This exothermic reaction consumes free water and produces hydration products. These products gradually fill the pores of the backfill, contributing to the development of a stable microstructure and a continuous increase in strength. The degree of cement hydration serves as a key parameter characterizing this process and is closely linked to the strength evolution of the material, as described by the following relationship^31^:

1 $$\:\left\{\begin{array}{l}\epsilon{(}{{t}}_{{e}}{)=}{\epsilon}_{{u}}\cdot\:{exp}\left[{-}{\left(\frac{\tau}{{{t}}_{{e}}}\right)}^\beta\right]\\\:{\epsilon}_{{u}}{=}\frac{{1.031}{{R}}_{{w}{/}{c}}}{{0.194+}{{R}}_{{w}{/}{c}}}{+0.5}{{X}}_{{FA}}{+0.3}{{X}}_{{slag}}\\\:{{t}}_{{e}}{=}{\int\:}_{{0}}^{{t}}{exp}\left[\frac{{{E}}_{{a}}}{{R}}\left(\frac{{1}}{{{T}}_{{r}}}{-}\frac{{1}}{{T}}\right)\right]{d}{t}\end{array}\right.$$

Within the model, the parameter $\:{\epsilon}_{{u}}$ denotes the ultimate degree of hydration. $\:{R}$ is the universal gas constant, set at a fixed value of $\:{8.314\hspace{0.33em}}{J}\cdot{mo}{{l}}^{{-1}}\cdot\:{{K}}^{{-1}}$. The water-to-cement ratio is represented by $\:{{R}}_{{w}{/}{c}}$, while $\:{{X}}_{{FA}}$ stands for the proportion of fly ash in the total binder mass. Additionally, $\:{{E}}_{{a}}$ corresponds to the apparent activation energy, expressed in units of J/mol.

### Thermal process

Following placement into the stope, the backfill slurry enters its curing stage, during which thermal interactions occur with the surrounding rock. Simultaneously, the hydration of cementitious components releases substantial heat, driving an internal temperature evolution. The associated heat-transfer mechanisms directly influence the progression of hydration and the self‑desiccation process, thereby affecting the overall reaction kinetics. In general, the thermal response of backfill can be attributed to two primary contributions: internal heat generation from the exothermic binder reaction, and heat redistribution via convection and conduction.

#### Heat generation

The dominant thermal source in cemented backfill is the hydration of cementitious materials, whose intensity—and consequent heat release—diminishes gradually over the curing period. The rate of heat production per unit volume can be expressed as:

2 $$\:\left\{\begin{array}{c}{{Q}}_{{hydr}{}}{=}{{H}}_{{T}}{\left(\frac{\tau}{{{t}}_{{e}}}\right)}^\beta\cdot\:\left(\frac\beta{{{t}}_{{e}}}\right)\cdot\epsilon\left({{t}}_{{e}}\right)\cdot\:{exp}\left[\frac{{E}_{a}}{{R}}\left(\frac{{1}}{{{T}}_{{r}}}{-}\frac{{1}}{{T}}\right)\right]\\\:{{H}}_{{T}}{=}\left({{H}}_{{cem}{}}\cdot\:{{X}}_{{cem}{}}{+461}{{X}}_{{slag\:}}{+1800}{{x}}_{{CaO}{/FA}}\right){{C}}_{{b}}\\\:{{H}}_{{cem}{}}{=500}{{x}}_{{{C}}_{{3}}{S}}{+260}{{x}}_{{{C}}_{{2}}{S}}{+866}{{x}}_{{{C}}_{{3}}{A}}{+420}{{x}}_{{{C}}_{{4}}{AF}}{+}\\\:{\hspace{1em}624}{{x}}_{{S}{{O}}_{{3}}}{+1186}{{x}}_{{FreeCaO}{}}{+850}{{x}}_{{MgO}}\end{array}\right.$$

The volumetric heat source arising from hydration is denoted by $\:{{Q}}_{{hydr}{}}$, while $\:{{H}}_{{T}}$ characterizes the total releasable heat of the cementitious system. The heat attributed solely to cement hydration is quantified by $\:{{H}}_{{cem}{}}$. The variable $\:\tau$ indicates the elapsed hydration time, and $\:{{t}}_{{e}}$ is the equivalent age accounting for temperature effects. The extent of the reaction is measured by the degree of hydration, $\:\epsilon$, with the shape of its evolution curve influenced by the parameter $\beta$. The kinetics of the reaction are governed by the apparent activation energy $\:{{E}}_{{a}}$. The universal gas constant is $\:{R}$. The instantaneous temperature of the material is $\:{T}$, which is evaluated relative to a specified reference temperature $\:{{T}}_{{r}}$. The proportions of cement and slag within the total binder are given by the mass fractions $\:{{X}}_{{cem}{}}$and $\:{{X}}_{{slag\:}}$, respectively. The apparent density of the cementitious matrix is $\:{{C}}_{{b}}$. For detailed heat calculation, $\:{{x}}_{{i}}$ defines the mass ratio of an individual mineral component $\:{i}$ to the total cement mass.

#### Thermal convection

Heat transfer due to convection in the backfill material can be expressed by the following set of Eqs^12–16^. :

3 $$\:{{Q}}_{{ad}}{=}\left({\rho}_{{w}}{{C}}_{{w-p}}{{v}}^{{rw}}{+}{\rho}_{{a}}{{C}}_{{a-p}}{{v}}^{{ra}}\right)\cdot{grad}\left({T}\right)$$

Here, $\:{{Q}}_{{ad}}$ represents the convective heat flux. $\:{\rho}_{{w}}$ and $\:{\rho}_{{a}}$ denote the densities of pore water and pore air, respectively. $\:{{v}}^{{ra}}$ and $\:{{v}}^{{rw}}$ are the Darcy velocities of water and air. $\:{{C}}_{{w}{-}{p}}$ and $\:{{C}}_{{a}{-}{p}}$ refer to the constant-pressure specific heat capacities of pore water and pore air.

#### Thermal conduction

After being placed into the stope, the backfill experiences thermal exchange with both the surrounding rock mass and the ambient curing conditions, primarily via conductive heat transfer. Detailed formulations and numerical treatments of this process can be found in references^29,32,33^.

4 $$\:\left\{\begin{array}{c}{q}{=-}{{k}}_{{eff\:}}{grad}\left({T}\right)\\\:{{k}}_{{eff\:}}{=}{{k}}_{{dry\:}}{+}\sqrt{{{S}}_{{eff\:}}}\left({{k}}_{{sat\:}}{-}{{k}}_{{dry\:}}\right)\\\:{{k}}_{{sat\:}}{=}{{k}}_{{tailings\:}}^{{1-}\theta}{{k}}_{{w}}^{\theta}\\\:{{k}}_{{dry\:}}{=}{{k}}_{{tailings\:}}^{{1-}\theta}{{k}}_{{a}}^{\theta}\end{array}\right.$$

In this formulation, the thermal conduction flux is represented by $\:{q}$, determined by the effective thermal conductivity $\:{{k}}_{{eff\:}}$ of the backfill. This effective conductivity depends on the respective values in the saturated state $\:{{k}}_{{sat\:}}$and the dry state $\:{{k}}_{{dry\:}}$, weighted by the effective saturation $\:{{S}}_{{eff\:}}$. The parameter $\:{{k}}_{{tailings\:}}$denotes the average thermal conductivity of the solid tailings particles, $\:{{k}}_{{w}}$ and $\:{{k}}_{{a}}$ are the conductivities of the pore water and air, respectively, and $\:\theta$ is the material’s porosity.

### Hydrological process

The hydrological process governing backfill materials encompasses fluid seepage as well as temporal changes in matric suction. The pore fluid consists of both water and air, whose movement through the porous matrix can be modeled using Darcy’s law.

5 $$\:{{v}}^{{ri}}{=-k}\frac{{{k}}_{{ri}}}{{\mu}_{{i}}}{grad}\left({{P}}_{{i}}{-}{\rho}_{{i}}{g}\right)$$

Here, the intrinsic permeability of the backfill matrix is given by $\:k$. For each fluid phase (water or air), $\:{{K}}_{{ri}}$ is its relative permeability, $\:{\mu}_{{i}}$ its dynamic viscosity, and $\:{{P}}_{{i}}$ its pressure. The symbol $\:{\rho}_{{i}}$ stands for density (with subscript $\:{i}$ specifying air, water, or solid), and $\:{g}$ is the acceleration due to gravity.

### Mechanical process

Chemical shrinkage and microstructural changes occur within the backfill as free water is consumed by ongoing cement hydration (a phenomenon known as self-desiccation). The overall deformation response, or total strain ($\:{\epsilon}$), arises from the superposition of several distinct mechanisms. These include strains from elastic deformation ($\:{{\epsilon}}_{{e}}$), irreversible plastic deformation ($\:{{\epsilon}}_{{p}}$), volume changes due to temperature variations ($\:{{\epsilon}}_{{T}}$), and strains induced by chemical reactions ($\:{{\epsilon}}_{{c}}$). Accordingly, the complete constitutive relationship can be expressed as:

6 $$\:{\epsilon=}{{\epsilon}}_{{e}}{+}{{\epsilon}}_{{p}}{+}{{\epsilon}}_{{r}}{+}{{\epsilon}}_{{T}}$$

### Multi-physics conservation equations

Although the individual mathematical models for the thermal, hydrological, mechanical, and chemical behaviors of backfill have been established, the complex interactions among these fields necessitate a coupled approach. Numerous researchers have developed various multi-field coupled models, such as Thermal-Chemical (TC), Thermal-Hydrological-Chemical (THC), and Thermal-Chemical-Mechanical (TCM) models, to predict specific properties like temperature evolution, pore water pressure, or strength development^17–19,27,28^. However, these existing models often fail to comprehensively account for the full Thermo-Hydro-Mechanical-Chemical (THMC) coupling effects, limiting their predictive capability and accuracy under complex conditions. To address these deficiencies, Cui and Fall^12,14–16^ developed a comprehensive THMC coupled model for backfill. This fully coupled framework integrates four governing equations—mass conservation of pore water (Eq. (7)) and pore air (Eq. (8)), momentum conservation (Eq. (9)), and energy conservation (Eq. (10))—thereby providing a more rigorous theoretical basis for simulating the multiphysics behavior of backfill.

$$\:\theta{S}\frac{{\partial}{\rho}_{{w}}}{{\partial}{t}}+\theta{\rho}_{{w}}\frac{{\partial}{S}}{{\partial}{t}}{+}{S}{\rho}_{{w}}\left[\frac{{\partial}{{\epsilon}}_{{v}}}{{\partial}{t}}{+}\frac{\left({1-\theta}\right)}{{\rho}_{{s}}}\frac{{\partial}{\rho}_{{s}}}{{\partial}{t}}\right]{-}{div}\left[\theta{S}{\rho}_{{w}}{k}\frac{{{k}}_{{rw}}}{{\mu}_{{w}}}\cdot{grad}\left({{P}}_{{w}}{-}{\rho}_{{w}}{g}\right)\right]{=}\theta{S}\left(\frac{{\rho}_{{w}}}{{\rho}_{{s}}}{S}{-1}\right)$$

7 $$\:\times\left\{{2}{{m}}_{{hc0}}\left({0.187}{{x}}_{{{C}}_{{s}}{S}}{+0.158}{{x}}_{{{C}}_{{s}}{S}}{+0.665}{{x}}_{{{C}}_{{s}}{A}}{+0.2130}{{x}}_{{{C}}_{{s}}{AF}}\right)\left\{{\left(\frac{\tau}{{{t}}_{{e}}}\right)}^\beta\left(\frac\beta{{{t}}_{{e}}}\right){\epsilon{exp}}\left[\frac{{{E}}_{{a}}}{{R}}\left(\frac{{1}}{{273+}{{T}}_{{r}}}{-}\frac{{1}}{{273+T}}\right)\right]\right\}\right\}$$

$$\:\theta\left({1-}{S}\right)\frac{{\partial}{\rho}_{{a}}}{{\partial}{t}}{-}\theta{\rho}_{{a}}\frac{{\partial}{S}}{{\partial}{t}}{+}\left({1-}{S}\right){\rho}_{{a}}\left[\frac{{\partial}{{\epsilon}}_{{v}}}{{\partial}{t}}{+}\frac{\left({1-\theta}\right)}{{\rho}_{{s}}}\frac{{\partial}{\rho}_{{s}}}{{\partial}{t}}\right]{-}{div}\left[\theta\left({1-}{S}\right){\rho}_{{a}}{k}\frac{{{k}}_{{r}{a}}}{{\mu}_{{a}}}\cdot{grad}\left({{P}}_{{a}}{-}{\rho}_{{a}}{g}\right)\right]{=}\left({1-}{S}\right)\theta{S}\frac{{\rho}_{{a}}}{{\rho}_{{s}}}$$

8 $$\:\times\left\{{2}{{m}}_{{hc0}}\left({0.187}{{x}}_{{{C}}_{{3}}{S}}{+0.158}{{x}}_{{{C}}_{{2}}{S}}{+0.665}{{x}}_{{{C}}_{{3}}{A}}{+0.2130}{{x}}_{{{C}}_{{4}}{AF}}\right)\left\{{\left(\frac{\tau}{{{t}}_{{e}}}\right)}^\beta\left(\frac\beta{{{t}}_{{e}}}\right){\epsilon{exp}}\left[\frac{{{E}}_{{a}}}{{R}}\left(\frac{{1}}{{273+}{{T}}_{{r}}}{-}\frac{{1}}{{273+T}}\right)\right]\right\}\right\}$$

9 $$\:{div}\left(\frac{{\partial}{\sigma}}{{\partial}{t}}\right){+}\frac{{\partial}\left[\left({1-}\theta\right){\rho}_{{s}}{+}\theta{S}{\rho}_{{w}}{+}\theta\left({1-}{S}\right){\rho}_{{a}}\right]}{{\partial}{t}}{g}{=0}$$

$$\:\left[\left(\frac{{1}}{{1+}{e}}\right){\rho}_{{s}}{{C}}_{{s}}{+}\frac{{e}}{{1+}{e}}{S}{\rho}_{{w}}{{C}}_{{w}}{+}\frac{{e}}{{1+}{e}}\left({1-}{S}\right){\rho}_{{a}}{{C}}_{{a}}\right]\frac{{\partial}{T}}{{\partial}{t}}{+}{div}\left({-}{{k}}_{{eff\:}}\cdot{grad}\left({T}\right)\right){+}\left({\rho}_{{w}}{{C}}_{{w}}{{v}}^{{rw}}{+}{\rho}_{{a}}{{C}}_{{a}}{{v}}^{{ra}}\right)\cdot{grad}\left({T}\right)$$

10 $$\:{=}\left({{H}}_{{c}}\cdot\:{{X}}_{{c}}{+461}{{X}}_{{slag}}{+1800}{{x}}_{{CaO}{/FA}}\cdot\:{{X}}_{{FA}}\right){{C}}_{{b}}{\left(\frac{\tau}{{{t}}_{{e}}}\right)}^\beta\left(\frac\beta{{{t}}_{{e}}}\right)\epsilon{exp}\left[\frac{{{E}}_{{a}}}{{R}}\left(\frac{{1}}{{273+}{{T}}_{{r}}}{-}\frac{{1}}{{273+T}}\right)\right]$$

where $\:{\rho}_{{s}}$ is the solid density, $\:{S}$ is the saturation, $\:{{\epsilon}}_{{v}}$is the volumetric strain, $\:{k}$ is the intrinsic permeability of the cemented tailings backfill, $\:{{k}}_{{rw}}$and$\:{{k}}_{{ra}}$represent the relative permeabilities of pore water and pore air, while $\:{\mu}_{{w}}$ and $\:{\mu}_{{a}}$ indicate the corresponding dynamic viscosities. $\:{{P}}_{{w}}$ and$\:{{P}}_{{a}}$ stand for pore water pressure and pore air pressure, respectively, $\:{e}$ is the void ratio. $\sigma$ corresponds to the macroscopic total stress tensor. In terms of thermal properties, $\:{{C}}_{{s}}$, $\:{{C}}_{{w}}$, and $\:{{C}}_{{a}}$ represent the specific heat capacities of the solid, water, and air, respectively. Parameters related to cement hydration include $\:{{m}}_{{hc0}}$(initial cement mass), $\:{{H}}_{{c}}$(total heat released during hydration), and $\:{{X}}_{{c}}$(mass fraction of cement in the mixture).

## Numerical simulation strategy and operating condition design

To thoroughly investigate the spatiotemporal evolution laws of THMC multi-field coupling within CPB under layered filling technology, this study constructs a numerical model that accounts for a time-varying computational domain. This section will elaborate on the moving mesh strategy based on the Arbitrary Lagrangian-Eulerian (ALE) method, the handling mechanism for moving boundary conditions, and the design of simulation cases tailored to the characteristics of layered filling.

### ALE-based moving mesh filling simulation method

Conventional finite element simulations often employ the “element birth and death” technique to simulate the layered stacking of backfill. However, this method suffers from issues such as strong mesh size dependency and discontinuous gradients of physical quantities at interfaces between new and old elements. To address these limitations, this study abandons the traditional approach and adopts a deforming mesh technique based on the ALE description to accurately capture the continuous rise of the slurry surface and the dynamic process of intermittent layering.

Within the ALE framework, the boundaries of the computational domain $\Omega$ undergo geometric displacement over time, with mesh nodes moving independently of the material^34,35^. The initial model state is set as a very thin layer region at the bottom of the stope ($\:{{h}}_{{0}}$=0.1 m). As the simulation time progresses, the top boundary of the model undergoes stretching deformation in the vertical direction. To simulate the layered construction process of “filling-curing-refilling,” the vertical mesh movement velocity $\:{{v}}_{{mesh}}$ is defined as a piecewise function of time:

11 $$\:{{v}}_{{mesh}}{=}\left\{\begin{array}{cc}{{v}}_{{fill}}&\:{t}\in{{T}}_{{fill}}\\\:{0}&\:{t}\in{{T}}_{{Maintenance}}\end{array}\right.$$

Where$\:{}{{v}}_{{mesh}}$ is the mesh deformation velocity and $\:{{v}}_{{fill}}$ is the designed slurry rise rate.

The specific layered simulation procedure is as follows: the top surface continuously rises at the velocity $\:{{v}}_{{fill}}$ until it reaches the designed height $\:{{h}}_{{1}}$ of the first layer. During this process, the mesh topology remains unchanged but stretches uniformly in the vertical direction, with the newly generated volume representing the freshly injected slurry. Subsequently, the top surface velocity is set to $\:{{v}}_{{mesh}}$=0, and the model geometry remains fixed. At this stage, the backfill undergoes hydration reaction, pore pressure dissipation, and self-weight consolidation in a static state. After the intermission period ends, the top surface is reactivated, continuing to rise at the velocity $\:{{v}}_{{fill}}$ to simulate the second layer of slurry covering the partially solidified lower backfill layer.

### Implementation in COMSOL multiphysics

To implement the THMC governing equations in COMSOL Multiphysics, the equations were transformed into the coefficient-form PDE framework. The dependent variables include the hydration degree, pore water pressure, pore air pressure, displacement components, and temperature. The second-order time derivative coefficient was set to zero for all equations because the present problem is governed by first-order transient diffusion, seepage, heat transfer, hydration kinetics, and quasi-static mechanical equilibrium. The coefficients used in the PDE implementation are summarized in Table 1. Hydration degree was introduced as an internal state variable and was coupled with the hydraulic, thermal, and mechanical fields through the hydration heat source, chemical water consumption, porosity evolution, permeability evolution, and strength development.

12 $$\:{{e}}_{{a}}\frac{{{\partial}}^{{2}}{u}}{{\partial}{{t}}^{{2}}}{+}{{d}}_{{a}}\frac{{\partial}{u}}{{\partial}{t}}{+}{\triangledown}\cdot\left({-}{c}{\triangledown}{u}{-}{\alpha}{u}{+}{\gamma}\right){+}{\beta}\cdot{\triangledown}{u}{+}{au}{=}{f}$$

**Table 1:** Coefficients used in the coefficient-form PDE implementation of the THMC model.

Governing equation	Dependent variable $\:{u}$	$\:{{d}}_{{a}}$	$\:{c}$	$\alpha$	$\gamma$	$\beta$	$\alpha$	$\:{f}$
Hydration kinetics	$\:\epsilon$	1	0	0	0	0	0	$\:{{R}}_{{h}}$
Water mass balance	$\:{{P}}_{{w}}$	$\:\theta{S}\frac{{\partial}{\rho}_{{w}}}{{\partial}{{P}}_{{w}}}$ $\:+\theta{\rho}_{{w}}\frac{{\partial}{S}}{{\partial}{{P}}_{{w}}}$​	$\:\theta{S}{\rho}_{{w}}{k}\frac{{{k}}_{{rw}}}{{\mu}_{{w}}}$	0	$\:\theta{Sk}\frac{{{k}}_{{rw}}}{{\mu}_{{w}}}{g}$	0	0	$\:\theta{S}\left(\frac{{\rho}_{{w}}}{{\rho}_{{s}}}{S}{-1}\right){{C}}_{{h}}$ $\:{-}{S}{\rho}_{{w}}\left[\frac{{\partial}{{\epsilon}}_{{v}}}{{\partial}{t}}{+}\frac{{1-\theta}}{{\rho}_{{s}}}\frac{{\partial}{\rho}_{{s}}}{{\partial}{t}}\right]$
Air mass balance	$\:{{P}}_{{a}}$	$\:{\theta(1-}{S}{)}\frac{{\partial}{\rho}_{{a}}}{{\partial}{{P}}_{{a}}}$ $\:{-\theta}{\rho}_{{a}}\frac{{\partial}{S}}{{\partial}{{P}}_{{a}}}$​	$\:{\theta(1-}{S}{)}{\rho}_{{a}}{k}\frac{{{k}}_{{ra}}}{{\mu}_{{a}}}$	0	$\:{\theta(1-}{S}{)}{k}{g}$	0	0	$\:{(1-}{S}{)}\theta{S}\frac{{\rho}_{{a}}}{{\rho}_{{s}}}{{C}}_{{h}}$ $\:{-(}{1}{-}{S}{)}{\rho}_{{a}}\left[\frac{{\partial}{{\epsilon}}_{{v}}}{{\partial}{t}}{+}\frac{{1-\theta}}{{\rho}_{{s}}}\frac{{\partial}{\rho}_{{s}}}{{\partial}{t}}\right]$
Momentum balance	$\:{{u}}_{{m}}{=[}{{u}}_{{x}}{,}{{u}}_{{z}}{{]}}^{{T}}$	0	$\:{D}{(}\epsilon{,}{T}{,}{S}{)}$	0	$\:{D}{:(}{{\epsilon}}^{{T}}{+}{{\epsilon}}^{{c}}{+}{{\epsilon}}^{{p}}{)}$ $\:{+}{{P}}_{{eff}}{I}$	0	0	$\:{-}{\rho}_{{mix}}{g}$
Energy conservation	$\:{T}$	$\:{{C}}_{{eff}}$	$\:{{k}}_{{eff}}$	0	0	$\:{\rho}_{{w}}{{C}}_{{w}}{{v}}^{{rw}}$ $\:{+}{\rho}_{{a}}{{C}}_{{a}}{{v}}^{{ra}}$	0	$\:{{Q}}_{{h}}$

### Geometric models and boundary conditions

**Fig. 3:**
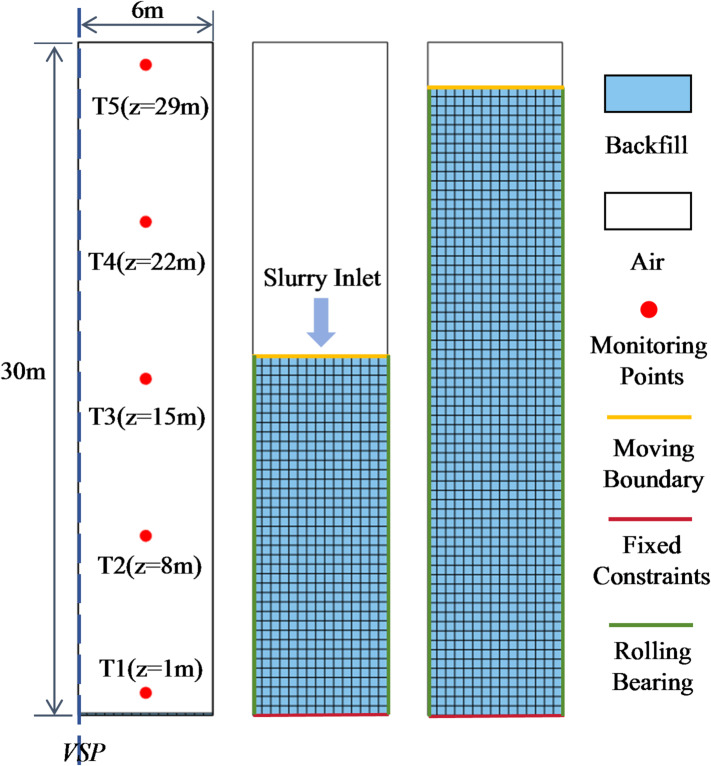
Schematic diagram of the geometric model.

Based on the symmetry characteristics of the stope structure and to optimize computational efficiency while ensuring accuracy, this study constructs a two-dimensional axisymmetric numerical model with a width of 6 m and a height of 30 m, as shown in Fig. 3.

In setting the boundary conditions, the model fully considers the coupling effects of mechanical, hydraulic, and thermal multi-physics fields. For the mechanical boundaries, both the left symmetry axis and the right contact surface with the surrounding rock are set as roller supports. This constrains horizontal displacement to simulate a laterally confined state while allowing free vertical settlement. The bottom boundary is treated as rigid bedrock with full constraints applied. To address the complex, time-dependent evolution of the computational domain during the layered filling process, the model top surface is defined as an evolving free surface. Considering the significant differences in environmental conditions between the filling and curing stages, the study implements dynamic switching of the top boundary conditions using a weak contribution form. Specifically, in the hydraulic field, the top boundary is set as a zero pore water pressure boundary during the filling period to simulate slurry filling and drainage. Upon entering the curing period, this pressure boundary is removed to simulate a closed consolidation environment. In the thermal field, the left, right, and bottom boundaries of the model are all set to unconstrained states (i.e., adiabatic boundaries). Meanwhile, the top boundary is constrained to the inlet slurry temperature of 20 °C during the filling period due to the continuous injection of fresh slurry. When entering the curing period, this temperature constraint is released, allowing the boundary to transition to a natural thermal evolution state. Furthermore, as the study employs moving mesh technology, the geometric topology of the computational domain is updated in real time via an automatic remeshing strategy as the filling height increases layer by layer. This method not only accurately captures the position of the continuously rising slurry surface but also effectively ensures mesh quality to avoid large deformation distortion, thereby achieving a dynamic and high-fidelity simulation of the backfill accumulation process.

### Simulated operating conditions design

To systematically reveal the spatiotemporal evolution mechanisms of the THMC multi-field coupling inside CPB under layered filling technology, this study formulates a numerical simulation scheme based on the aforementioned two-dimensional axisymmetric model. The fundamental physical and mechanical parameters for the simulation are set according to laboratory experiments and engineering field data. The hydraulic characteristics of the backfill slurry are described using the Van Genuchten model, with its initial permeability coefficient and model fitting parameters listed in Table 3. Regarding thermodynamic boundary conditions, a constant slurry inlet temperature and initial ground temperature of the surrounding rock are set, and a continuous filling operation process at a constant rate of $\:{{v}}_{{fill}}{=0.5}{m}{/h}$ is simulated.

**Table 2:** Calculation case design and parameter combination table.

Case ID	c/s ratios	Binder content	Layering Strategy	Single Filling Height/m	Interval Time/h
Case-Base	1:8	0.111	Three-layer	10	24
Case-M1	1:4	0.2	Three-layer	10	24
Case-M2	1:12	0.077	Three-layer	10	24
Case-S1	1:8	0.111	Continuous	30	0
Case-S2	1:8	0.111	Two-layer	15	24
Case-T1	1:8	0.111	Three-layer	10	12
Case-T2	1:8	0.111	Three-layer	10	72

**Table 3:** Model basic parameter Tables^12,14–16^.

Parameter	Unit	Value	Parameter	Unit	Value
Hydration shape parameter $\beta$	1	0.394	Thermal conductivity of water $\:{{k}}_{{w}}$	$\:{W}{/(}{m}\cdot{K}{)}$	0.58
Specific volume of chemically bound water $\:{{v}}_{{c}{w}}$	$\:{{cm}}^{{3}}{/}{g}$	0.72	Thermal conductivity of tailings $\:{{k}}_{{tailings\:}}$	$\:{W}{/(}{m}\cdot{K}{)}$	1.38
Specific volume of adsorbed water $\:{{v}}_{{ab}{-}{w}}$	$\:{{cm}}^{{3}}{/}{g}$	0.9	Specific heat capacity of tailings $\:{{C}}_{{s}{-}{p}}$	$\:{J}{/(}{kg}\cdot{K}{)}$	850
Specific volume of water$\:{{}{v}}_{{w}}$	$\:{{m}}^{{3}}{/}{kg}$	1 × 10^− 3^	Specific heat capacity of water $\:{{C}}_{{w}{-}{p}}$	$\:{J}{/(}{kg}\cdot{K}{)}$	4180
Specific volume of hydration products$\:{}{{v}}_{{n}}$	$\:{{m}}^{{3}}{/}{kg}$	7.2 × 10^− 4^	Saturated permeability coefficient $\:{{k}}_{{s}{0}}$	$\:{m}{/}{s}$	1 × 10^− 6^
Specific volume of cement particles $\:{{v}}_{{c}}$	$\:{{m}}^{{3}}{/}{kg}$	3.2 × 10^− 4^	VG model fitting parameter $\alpha$	$\:{1/}{m}$	2.06
Specific volume of tailings particles$\:{}{{v}}_{{tailings}}$	$\:{{m}}^{{3}}{/}{kg}$	3.8 × 10^− 4^	VG model fitting parameter $\:{n}$	1	1.69
Hydration time parameter$\:{}\tau$	$\:{h}$	33.6	Initial porosity $\:{\theta}_{{0}}$	1	0.472
$\:{{x}}_{{{C}}_{{3}}{S}}$	1	0.232	$\:{{x}}_{{{C}}_{{2}}{S}}$	1	0.462
$\:{{x}}_{{{C}}_{{3}}{A}}$	1	0.087	$\:{{x}}_{{{C}}_{{4}}{AF}}$	1	0.096
$\:{{x}}_{{S}{{O}}_{{3}}}$	1	0	$\:{{x}}_{{FreeCaO}{}}$	1	0
$\:{{x}}_{{MgO}}$	1	0	$\:{{X}}_{{slag\:}}$	1	0
$\:{{x}}_{{CaO}{/FA}}$	1	0	Apparent density of the cementitious matrix $\:{{C}}_{{b}}$	$\:{{kg/m}}^{{3}}$	157.23

Building upon the baseline case, the study conducts sensitivity analyses on material mix proportions and construction process parameters, respectively. For material mix proportions, three groups of different cement-to-sand ratio gradients are set by adjusting the mass fraction of cementitious materials, aiming to investigate the driving effects of different ratios on the hydration reaction and consolidation process. The specific cement-to-sand ratios and binder contents for each group are detailed in Table 2. Regarding process parameters, the study focuses on examining the coupling effect between the layering structure and intermittent timing. The layering structure design encompasses various modes from continuous filling to multi-layer filling, aiming to quantify the impact of the number of layers on pore pressure dissipation and strength development. Simultaneously, different durations of inter-layer intervals are set to reveal the heat-water transfer laws at interfaces with different consolidation ages. The specific parameter combinations for each simulated case and the fundamental physical parameters are detailed in Tables 2 and 3.

## Model validation

To verify the reliability and accuracy of the developed THMC coupling model for layered backfill, this study selected the well-documented high column experiments conducted by Ghirian and Fall^29,30^ as a benchmark case. These experiments provide comprehensive monitoring data on the multi-field evolution of CPB under staged backfilling conditions, which aligns perfectly with the scope of this research.

**Fig. 4:**
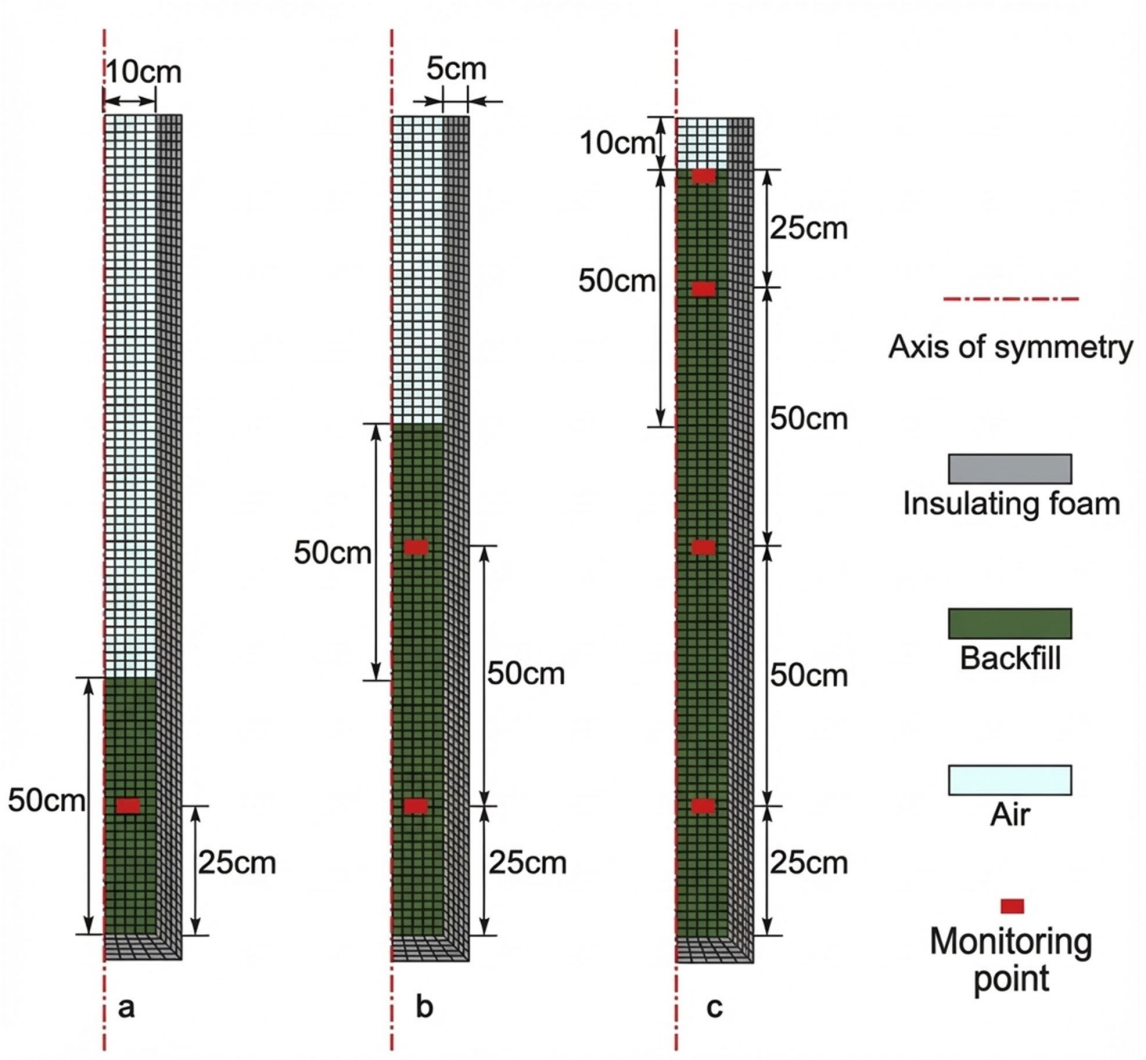
Schematic diagram of the Ghirian and Fall layered backfilling experimental model^29,30^.

The reference experiment involved a CPB column with a height of 150 cm and a diameter of 10 cm. The backfilling was performed in three stages (layers), with each layer being 50 cm high. A curing interval of 24 h was set between each layer to simulate the layered construction process. The experimental setup included insulating foam to create semi-adiabatic conditions, and sensors were embedded at three different heights (Bottom: 25 cm, Middle: 75 cm, Top: 125 cm) to monitor temperature and pore water pressure (PWP) evolution. Corresponding to the experiment, an axisymmetric numerical model was established. The geometry, mesh generation, and monitoring point locations are illustrated in Fig. 4. The boundary conditions were set to match the insulated-undrained conditions of the physical experiment.

**Fig. 5:**
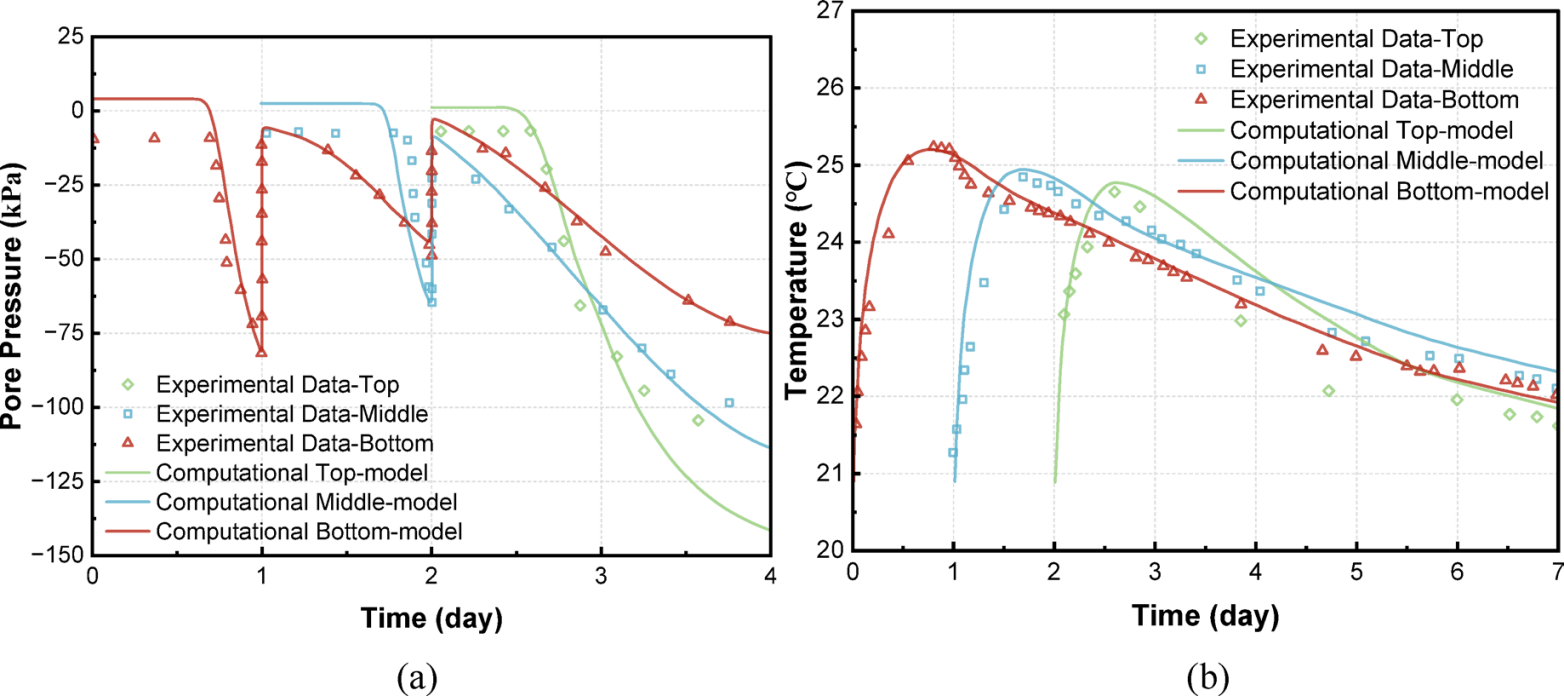
Comparison curves between experimental and numerical simulation results. (**a**) Pore water pressure curves at monitoring points. (**b**) Temperature curves at monitoring points.

The evolution of PWP is a critical indicator of the consolidation process. Figure 5(a) compares the simulated PWP with the experimental measurements. The results show that the model successfully reproduces the complex hydraulic response induced by staged backfilling. Notably, the model captures the “suction” phenomenon (negative PWP) developed during the curing intervals and the transient PWP spikes caused by the placement of subsequent fresh backfill layers. The consistency between the simulation and experiment confirms the model’s capability in coupling the hydraulic flow with mechanical deformation and hydration processes.

Figure 5(b) presents the comparison between the numerical simulation results and the experimental data regarding the temperature evolution at three monitoring points. It can be observed that the model accurately captures the hydration heat generation and dissipation process. Specifically, the peak temperatures and their occurrence times for all three layers are well-predicted. The rapid temperature rise in the early stage due to binder hydration and the subsequent gradual decrease are consistent with the measured data. The high agreement indicates that the proposed model effectively describes the heat transfer and heat source terms in the layered CPB.

To further quantify the agreement between the experimental measurements and numerical simulations, an error analysis was conducted using the mean absolute error (MAE), root mean square error (RMSE), and coefficient of determination (R^2^). The results are summarized in Table 4.

**Table 4:** Error analysis between experimental and numerical results.

Variable	Position	MAE	RMSE	*R* ^2^
Temperature	Top	0.328	0.421	0.827
	Middle	0.255	0.374	0.863
	Bottom	0.129	0.197	0.968
Pore pressure	Top	7.581	10.990	0.836
	Middle	3.846	6.640	0.897
	Bottom	8.087	9.970	0.930

As shown in Table 4, the numerical simulations agree well with the experimental measurements. For temperature, the MAE ranges from 0.129 to 0.328, and the RMSE ranges from 0.197 to 0.421. The corresponding R^2^ values are between 0.827 and 0.968, indicating that the numerical model can accurately reproduce the measured temperature response at different positions. For pore pressure, the MAE ranges from 3.846 to 8.087, and the RMSE ranges from 6.640 to 10.990. Although the pore pressure errors are larger than those of temperature, the R^2^ values remain relatively high, ranging from 0.836 to 0.930. This indicates that the numerical simulations can reasonably capture the overall variation trend of pore pressure, despite some local deviations in magnitude.

Overall, the numerical predictions show excellent agreement with the experimental data for both thermal and hydraulic fields, demonstrating the robustness of the developed THMC model in simulating the multi-physics behavior of layered CPB.

## Results

### Influence of of stratified filling strategies

**Fig. 6:**
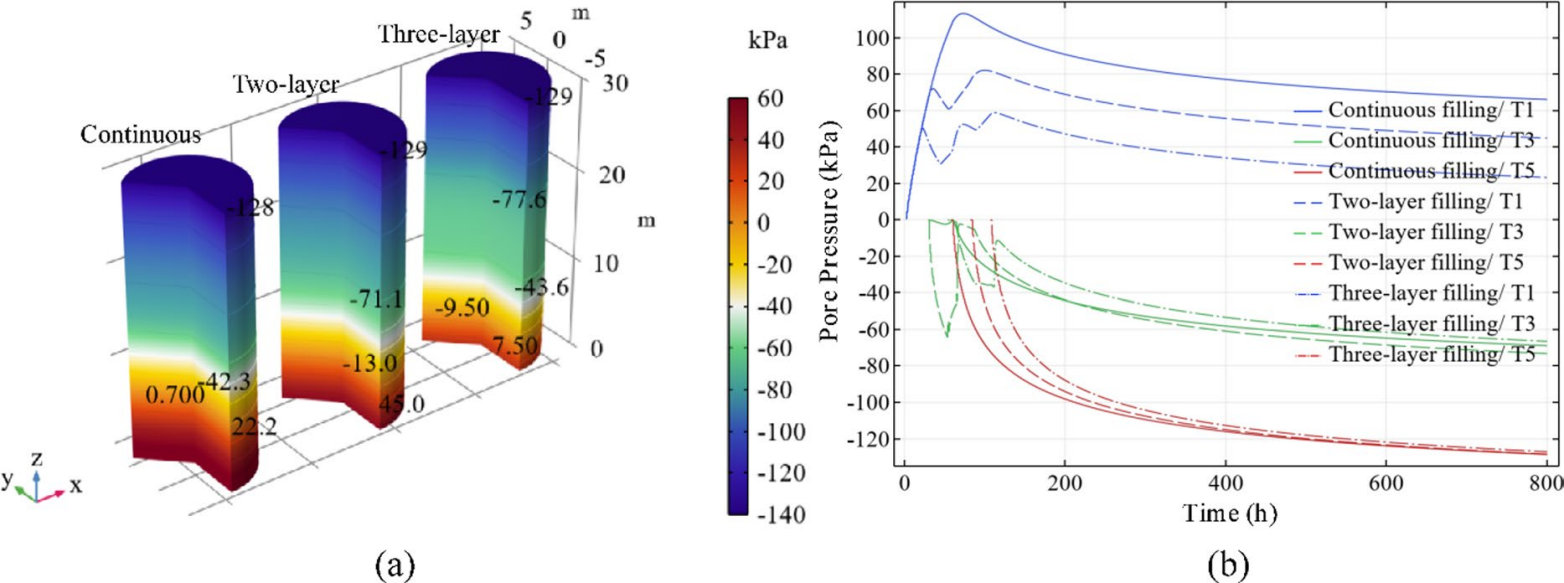
Spatiotemporal evolution of pore pressure in backfill under different layer counts. (**a**) Spatial distribution of pore pressure at 800 h. (**b**) Effect of the number of layers on pore pressure.

**Fig. 7:**
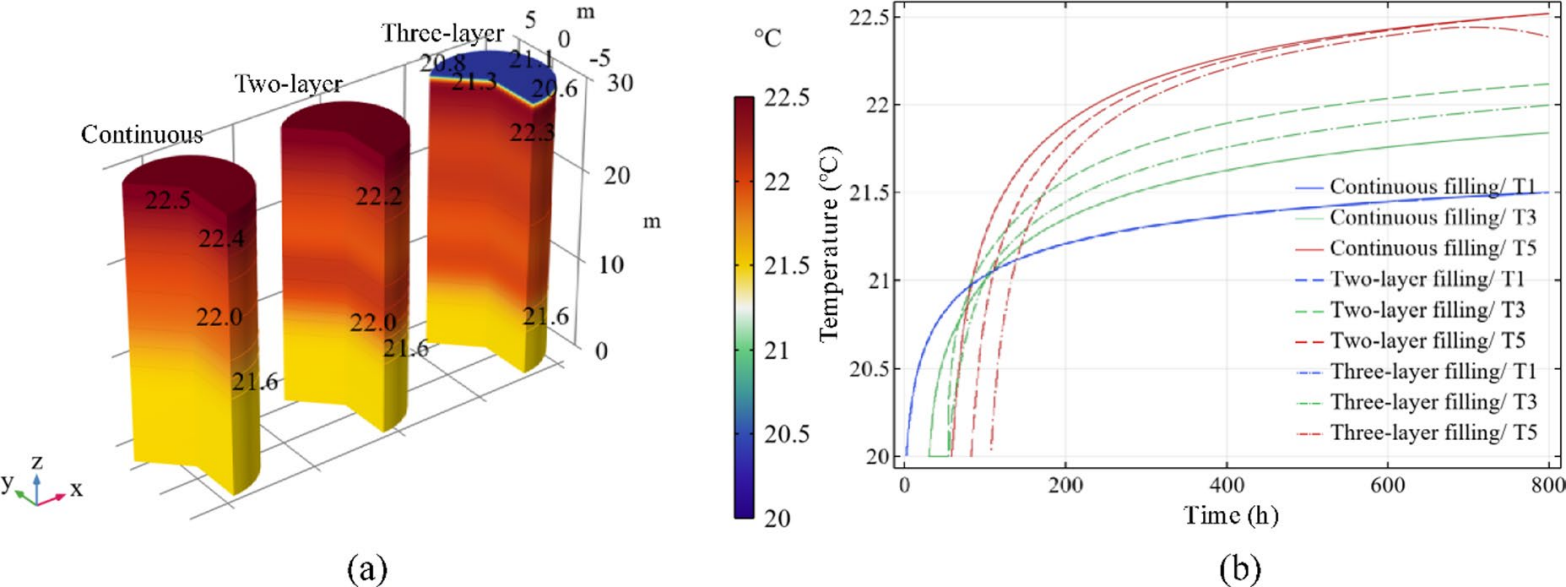
Spatiotemporal evolution of temperature in backfill under different layer counts. (**a**) Spatial distribution of temperature at 800 h. (**b**) Effect of the number of layers on temperature.

**Fig. 8:**
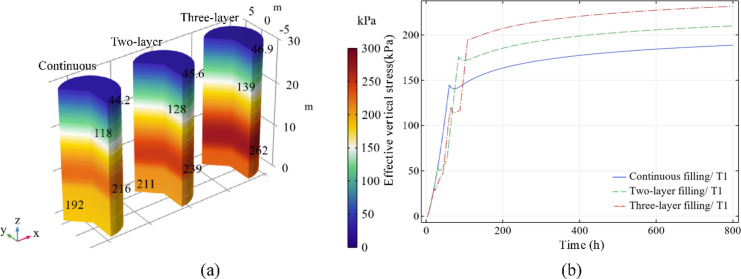
Spatiotemporal evolution of effective vertical stress in backfill under different layer counts. (**a**) Spatial distribution of effective vertical stress at 800 h, (**b**) Effect of the number of layers on effective vertical stress.

Figures 6, 7 and 8 provide an in-depth analysis of the regulatory mechanisms of layered filling strategies (Continuous, Two-layer, and Three-layer filling) on the spatiotemporal evolution of the multi-physics fields within the backfill. Similar to the influence of filling interval time, the number of layers directly determines the loading rate and boundary drainage conditions of the backfill, serving as a core process parameter for balancing filling efficiency with stability.

First, Fig. 6 visually demonstrates the decisive advantage of layered filling in controlling PWP. Under the continuous filling condition, the rapid, one-time pouring of slurry leads to a drastic accumulation of PWP due to the immense self-weight load. The blue curve in Fig. 6(b) shows the peak PWP exceeding 100 kPa with an extremely slow dissipation process, which poses a high risk of backfill liquefaction or excessive lateral pressure on the barricade. In contrast, as the number of layers increases (i.e., the height of a single layer decreases), the PWP evolution exhibits a distinct “sawtooth” dissipation characteristic. Particularly in the three-layer filling scenario, the interval after each pour acts as a “drainage window,” allowing excess pore pressure to be effectively released before the next layer is poured. The 800 h contour map in Fig. 6(a) further confirms that a widespread negative PWP zone (dark blue area) has formed within the three-layer backfill, indicating that matric suction is fully established and the backfill is in a highly stable state.

Furthermore, the influence of layer number on the temperature field is mainly reflected in the temporal distribution of heat (Fig. 7). Continuous filling results in a concentrated release of hydration heat within a short period; although the early temperature rise is rapid, the risk of heat accumulation is high. Layered filling, however, disperses the heat source introduction over different time periods. As shown in Fig. 7(b), this spatiotemporal difference in the temperature field suggests that in the design of layered filling, attention must be paid not only to mechanical stability but also to the thermal stress coupling effects between different layer interfaces.

Comparing the evolution curves in Fig. 8(b), although continuous filling shows smooth stress growth, its final effective stress is lower (approx. 180 kPa) due to the counteracting effect of high PWP. However, under the three-layer filling condition, the effective stress exhibits a “stepwise” growth pattern; each rapid dissipation of PWP is accompanied by a jump in effective stress, with the final effective vertical stress at point T1 exceeding 200 kPa. The spatial distribution in Fig. 8(a) also clearly shows that the high-stress core zone (dark red) at the bottom of the three-layer backfill is the most extensive and strongest. This indicates that the layered filling strategy, by “breaking up the whole into parts,” effectively avoids the stress lag effect caused by continuous filling, significantly improving the early consolidation strength and overall bearing capacity of the backfill. In summary, the three-layer filling scheme demonstrates optimal engineering adaptability in reducing peak PWP risks and enhancing effective stress.

### The effect of c/s ratios on the THMC of CPB

**Fig. 9:**
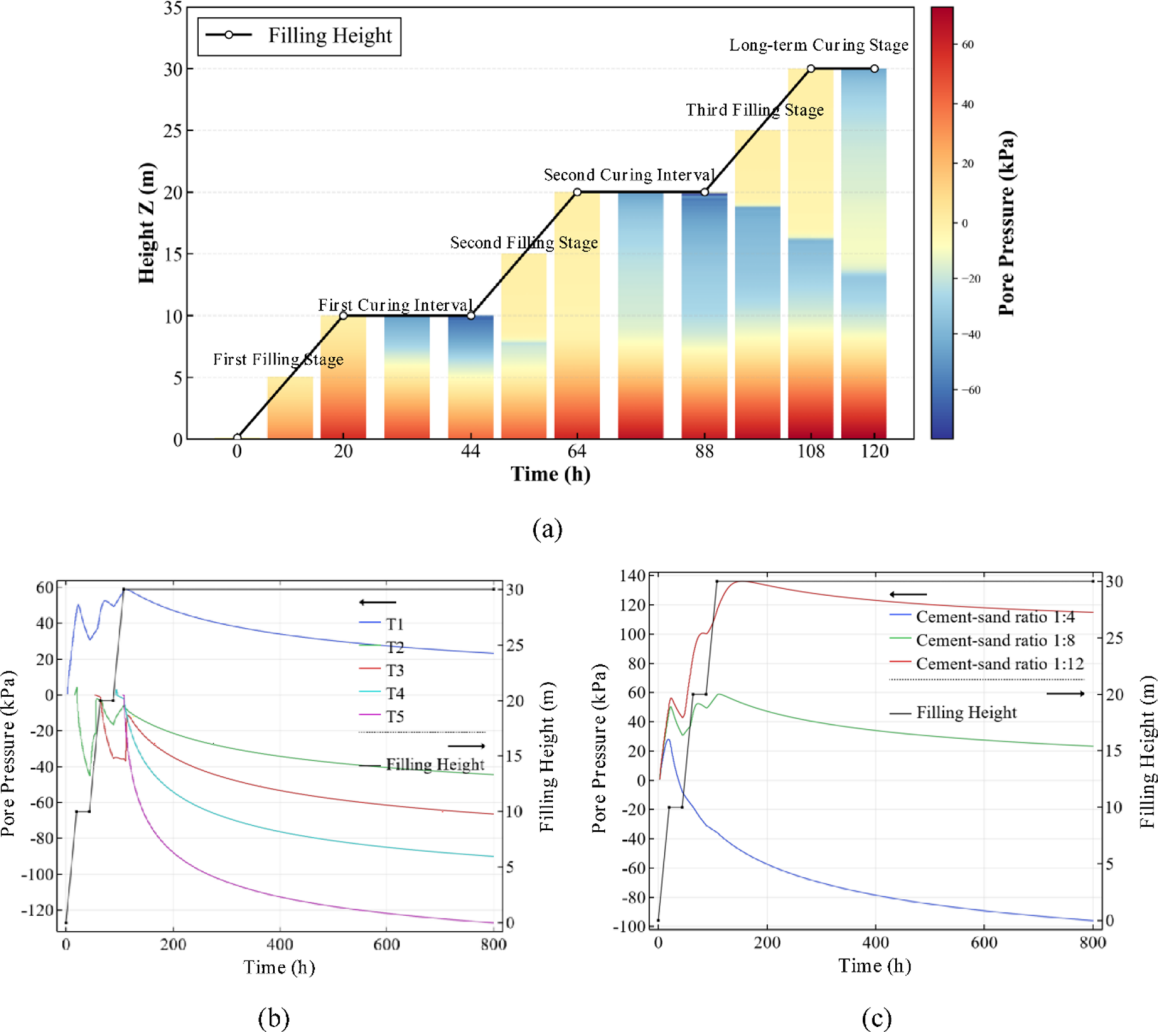
Spatiotemporal evolution of pore water pressure in backfill. (**a**) Spatial distribution characteristics of pore water pressure in the early filling stage. (**b**) Pore pressure evolution at various monitoring points. (**c**) Effect of c/s ratios on the PWP.

The spatiotemporal evolution of PWP serves as a critical indicator for evaluating the consolidation characteristics and stability of CPB, with the c/s ratios playing a dominant role in this process. As illustrated in Fig. 9, the PWP response exhibits distinct distinct phasic characteristics, highly synchronized with the “filling-curing” construction cycles. During the initial filling stages, the injection of fresh slurry induces an immediate surge in PWP at various monitoring points due to the increase in overburden total stress. However, as the curing intervals proceed, the dissipation paths of PWP diverge significantly among different c/s ratios.

As shown in Fig. 9(a), the PWP distribution during the early filling stage exhibits clear spatiotemporal heterogeneity governed by the staged filling process. The lower part of the backfill responds earlier because it is filled first, whereas the upper part remains inactive until the slurry reaches the corresponding elevation. During each filling stage, the increasing self-weight load induces a rapid rise in PWP. During the subsequent curing interval, PWP gradually dissipates, and negative PWP develops in the previously placed layer due to drainage and hydration-induced self-desiccation. Figure 9(b) further confirms that the PWP response at each monitoring point follows the filling sequence, with lower points responding earlier and upper points showing delayed responses.

The cement-sand ratio further modifies this staged PWP evolution. For the high cement-sand ratio of 1:4, PWP dissipates rapidly and changes from positive pressure to negative pressure at an early age, finally stabilizing at approximately − 100 kPa. This is mainly attributed to the stronger hydration reaction induced by the higher cement content, which consumes pore water and generates hydration products to refine the pore structure. Consequently, matric suction and effective stress develop more rapidly, improving the early self-supporting capacity of CPB.

By contrast, the low cement-sand ratio of 1:12 shows a much slower PWP dissipation. As shown in Fig. 9(c), the PWP remains positive under the same filling height, with a peak exceeding 120 kPa. This is because the low cement content results in weak hydration, limited chemical water consumption, and insufficient pore refinement, keeping the slurry close to saturation for a longer period. In this case, PWP is mainly governed by the hydrostatic pressure induced by self-weight, which reduces effective stress and increases the potential risk of liquefaction or barricade failure. The 1:8 case exhibits an intermediate response: its PWP decreases gradually but remains within a low positive range, indicating that hydration is still insufficient to fully counteract the PWP accumulation caused by self-weight.

**Fig. 10:**
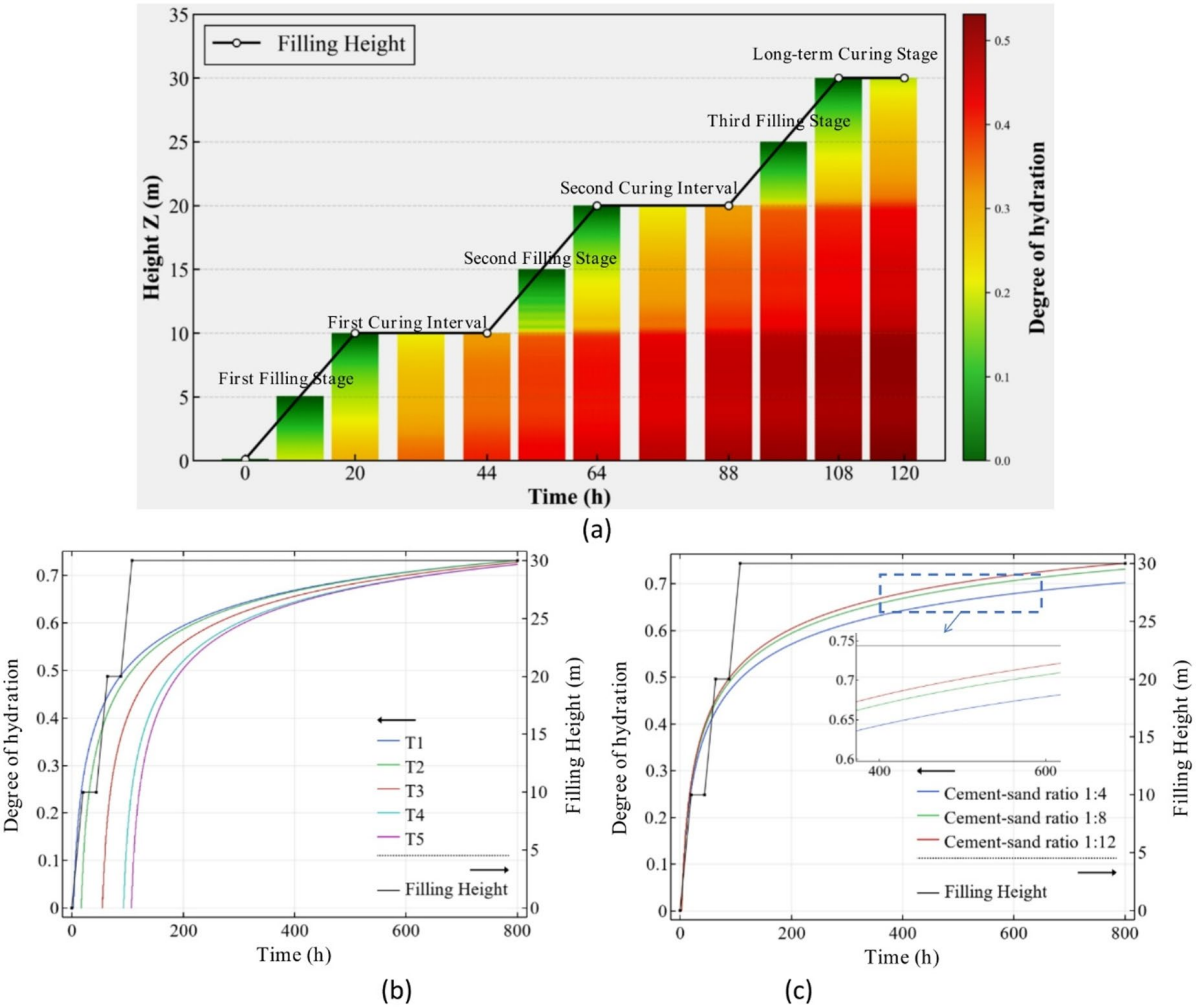
Spatiotemporal evolution of the degree of hydration in backfill. (**a**)Spatial distribution characteristics of the degree of hydration in the early filling stage, (**b**) Evolution history of the DoH at various monitoring points, (**c**) Effect of c/s ratios on the DoH.

Cement hydration constitutes the fundamental driving force behind the strength development and microstructural evolution of CPB, and its progression is significantly influenced by the c/s ratios. As illustrated in Fig. 10, the degree of hydration (DoH) of the backfill exhibits pronounced spatiotemporal heterogeneity and mix-dependence. Observing the spatial distribution characteristics in Fig. 10(a), regions filled earlier at the bottom (corresponding to monitoring points T1 and T2) exhibit a significantly higher degree of hydration compared to the newly filled upper regions. This gradient distribution reflects the impact of the construction sequence and implies that the deeper backfill acquires substantial cementation strength early on.

Figure 10(b) further reveals the evolutionary history of DoH at various monitoring points under the Base Case. The hydration degree at all points follows a typical kinetic characteristic of “rapid growth in the early stage, followed by gradual leveling off.” Although the final DoH values for points T1 through T5 tend to converge, there is a distinct lag in their onset times, strictly corresponding to the timeline of the staged filling construction. This temporal staggering implies significant stiffness disparity within the backfill at any given moment.

The influence of different cement-sand ratios on hydration kinetics is clearly elucidated in Fig. 10(c), presenting an inverse correlation that warrants careful analysis. Contrary to the intuitive assumption that higher cement content yields a higher degree of reaction, the results indicate that a higher cement-sand ratio leads to a lower ultimate degree of hydration. Specifically, the CPB with a 1:4 ratio (blue line) exhibits a noticeably lower DoH after 400 h compared to the 1:8 (green line) and 1:12 (red line) ratios. The mechanism underlying this phenomenon lies in the mix proportioning of the slurry. Under the condition of a constant mass concentration, increasing the cement-sand ratio increases the proportion of cement in the solid phase, which inevitably results in a significant reduction of the water-to-cement ratio (w/c). For the high c/s ratio (1:4) backfill, the amount of free water available per unit mass of cement is limited. Consequently, the hydration reaction becomes constrained by water availability (a “water starvation” effect) in the later stages, preventing the full hydration of clinker particles and restricting the further growth of DoH. Conversely, the low c/s ratio (1:12) backfill possesses a higher w/c ratio, providing ample water for the relatively fewer cement particles to react more completely, thereby achieving a higher ultimate degree of hydration.

**Fig. 11:**
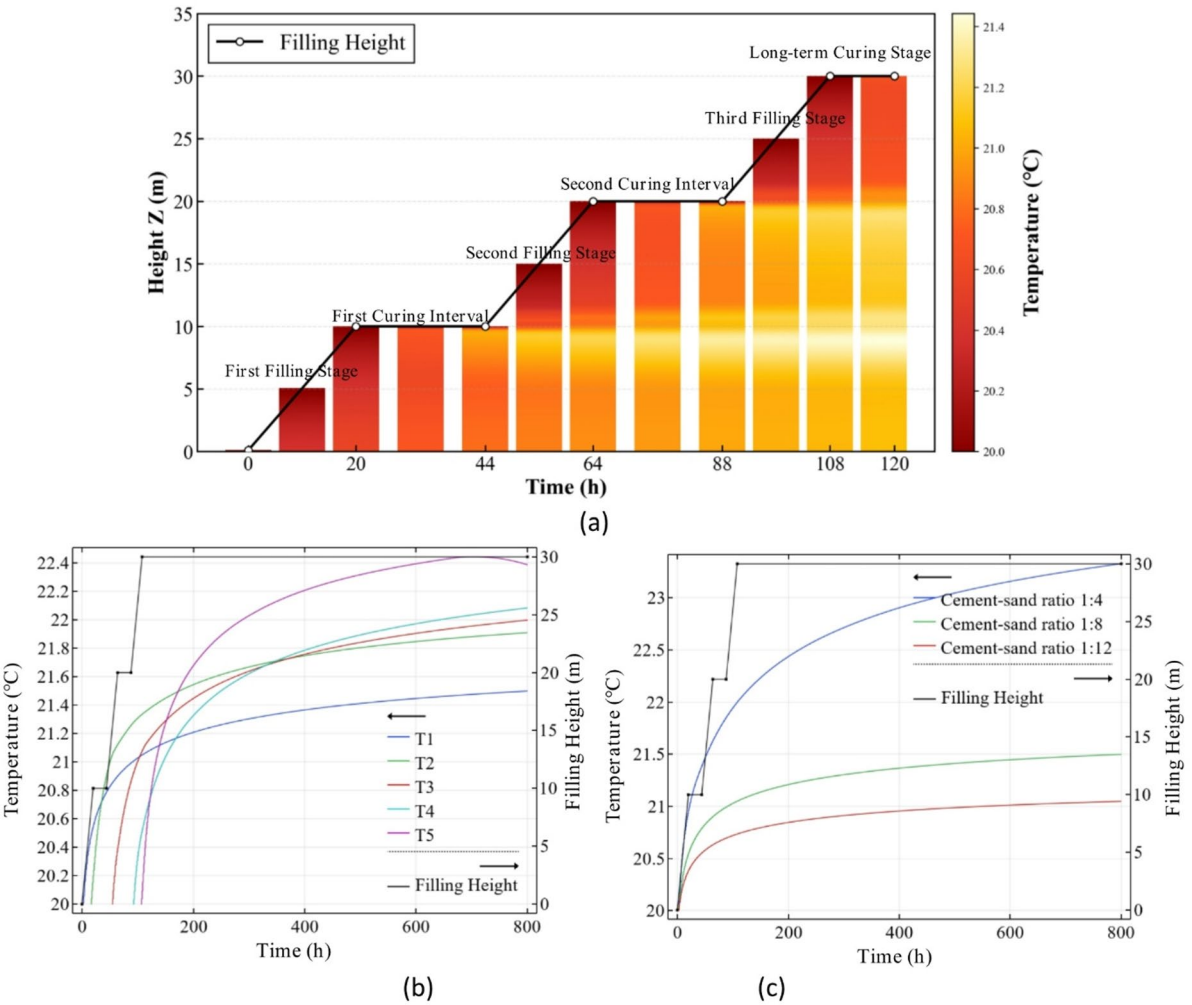
Spatiotemporal evolution of temperature in backfill. (**a**)Spatial distribution characteristics of temperature in the early filling stage, (**b**) Temperature evolution at various monitoring points, (**c**) Effect of c/s ratios on temperature change.

Figure 11 illustrates the dynamic characteristics of the backfill temperature over time and space. Since the model does not consider the surrounding rock boundary, the temperature evolution is primarily governed by the internal heat release from cement hydration and heat accumulation, closely correlating with the development of the DoH discussed previously.

Combining the contour plot in Fig. 11(a) with the monitoring curves in Fig. 11(b), the temperature field exhibits distinct stratified progression and heat accumulation. As the three filling stages proceed sequentially, the heat source (cement hydration) is superimposed layer by layer in space. The temperature rise curves for all monitoring points (T1-T5) exhibit a non-linear growth pattern of “rapid rise — gradual increase — stabilization.” The initiation times for points T1 through T5 strictly correspond to the construction sequence. Due to the fastest hydration rate in the early age, all points undergo intense temperature rise initially, followed by a gradual leveling off as the hydration rate decays. Figure 11(c) further reveals the significant influence of the c/s ratios on the temperature field. The graph shows a positive correlation between the temperature rise and the c/s ratio: the 1:4 ratio reaches the highest final temperature (approaching 23 °C), significantly exceeding that of the 1:8 and 1:12 ratios.

This phenomenon provides a critical physical complement to the previous analysis of DoH: Although it was noted that a high c/s ratio (1:4) limits the hydration efficiency of individual cement particles (lower DoH) due to a lower water-to-cement ratio, the results in Fig. 11(c) indicate that the volumetric cement content (total reactant mass) is the dominant thermodynamic factor determining the magnitude of temperature rise. The extremely high cement base in the 1:4 slurry results in a total heat release per unit volume that far surpasses that of low c/s slurries. This thermal effect, driven by the “advantage of total reactant quantity,” fully compensates for and overrides the negative impact of “reduced individual particle efficiency (DoH),” ultimately resulting in the highest macroscopic temperature for the high c/s backfill.

**Fig. 12:**
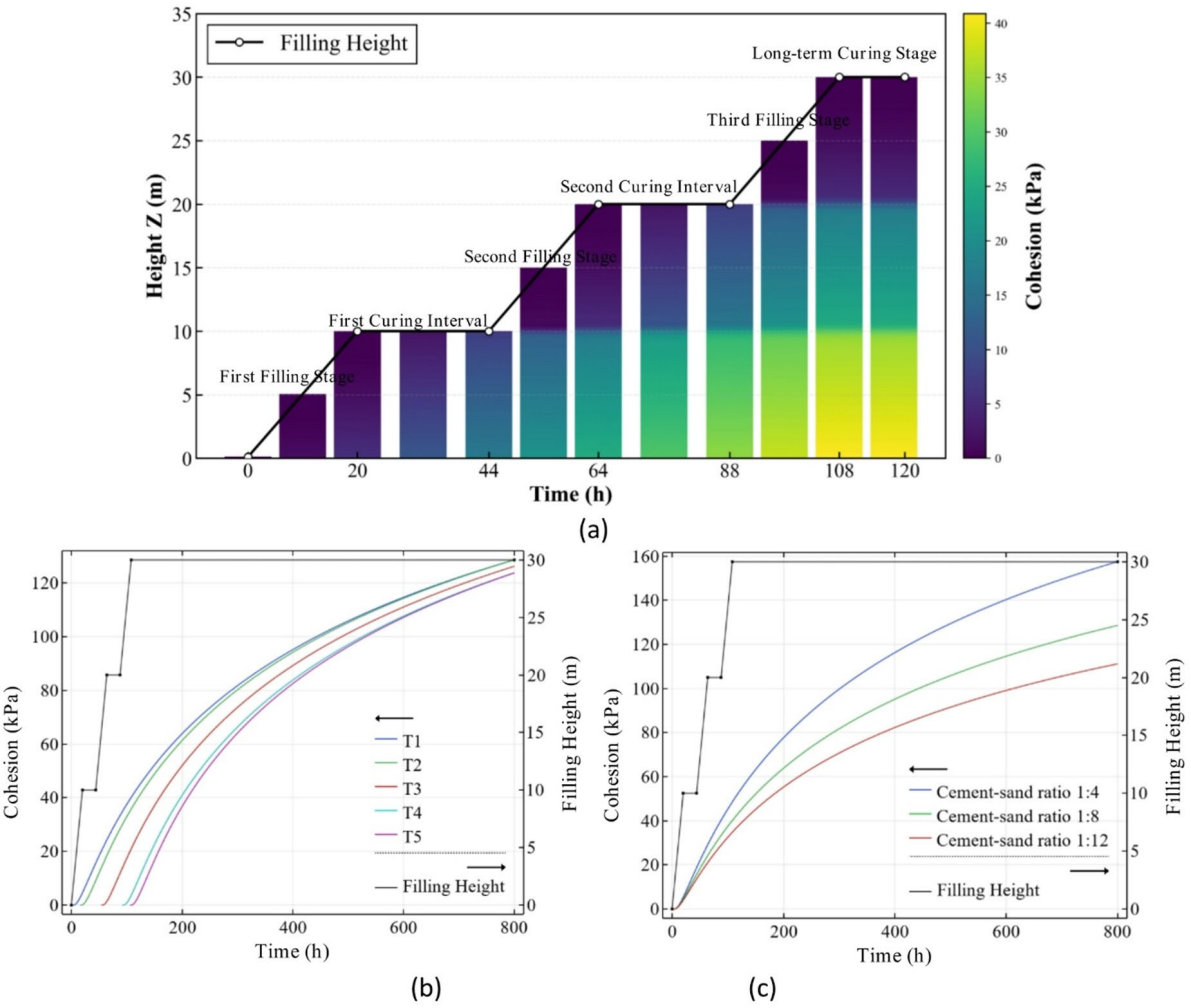
Spatiotemporal evolution of cohesion in backfill. (**a**)Spatial distribution characteristics of cohesion in the early filling stage, (**b**) Cohesion evolution at various monitoring points, (**c**) Effect of c/s ratios on cohesion change.

Figure 12 illustrates the evolutionary characteristics of backfill cohesion over time and space. As a direct mechanical product of the cement hydration reaction, the growth trend of cohesion shows intrinsic consistency with the accumulation process of the temperature field described previously. Combining the spatial distribution contour in Fig. 12(a) with the monitoring point curves in Fig. 12(b), the establishment of backfill strength demonstrates distinct spatiotemporal lag and stratified hardening characteristics. As the three filling stages progress sequentially, the cohesion at monitoring points (T1-T5) does not increase synchronously but initiates strictly according to the construction timeline of the staged filling. Point T1, having started consolidation earliest, exhibits the most fully developed strength, whereas point T5 at the top only begins to establish strength after the slurry reaches that elevation. In terms of curve shape, all points display a non-linear characteristic of “rapid growth—gradual slowing—stabilization.” This change in slope intuitively reflects the dynamic process where cementing materials are rapidly generated to form a skeleton within the backfill, followed by a gradual approach to a limit value as reactants are consumed.

Figure 12(c) further reveals the decisive influence of the cement-sand ratio on cohesion development. Similar to the pattern observed in the temperature field, the peak cohesion of the backfill is significantly positively correlated with the cement-sand ratio: the final cohesion of the 1:4 high ratio backfill approaches 160 kPa, far exceeding that of the 1:8 and 1:12 ratios. This indicates that the cement content per unit volume is the primary factor controlling the mechanical performance of the backfill. The high cement-sand ratio slurry possesses a larger cement base, capable of generating a more massive volume of gel products during hydration. These products effectively fill pores and strongly bind tailings particles, manifesting macroscopically as a significant enhancement in shear strength, thereby establishing the absolute advantage of high cement-sand ratios in both early strength establishment and ultimate mechanical performance.

### Influence of filling interval time

**Fig. 13:**
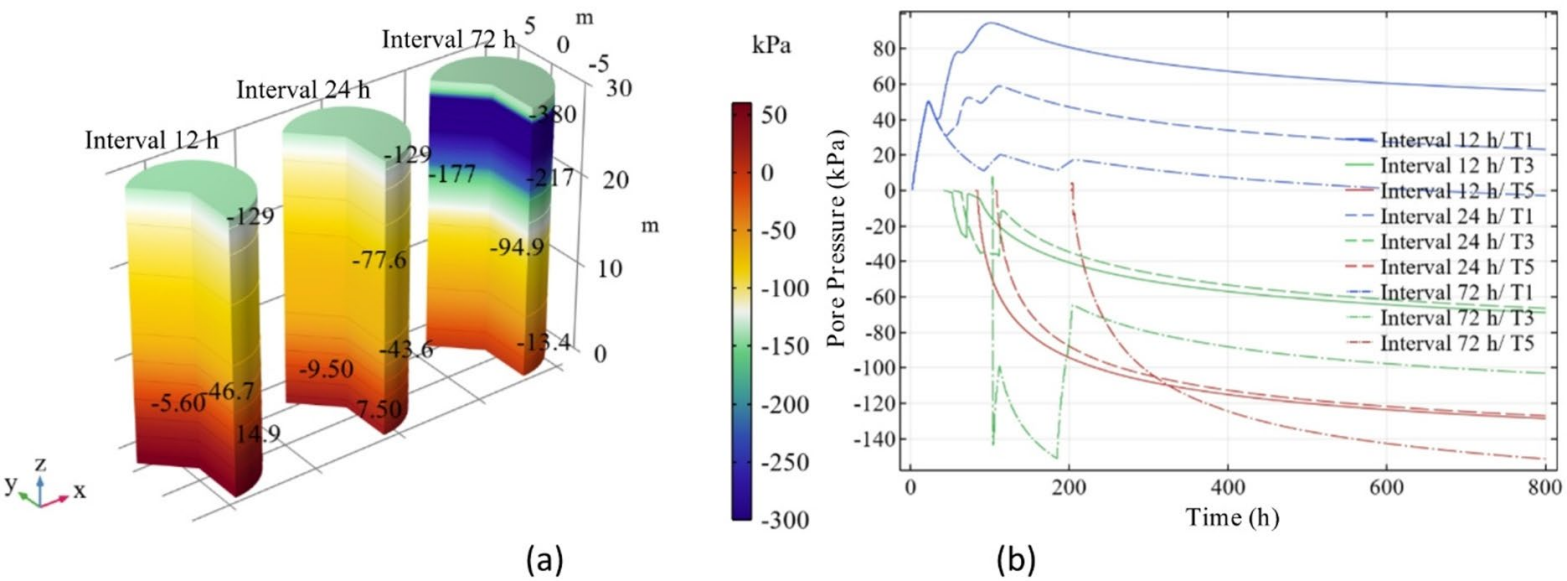
Spatiotemporal evolution of pore pressure in backfill under different filling interval times. (**a**)Spatial distribution of pore pressure at 800 h, (**b**) Effect of interval time on pore pressure.

**Fig. 14:**
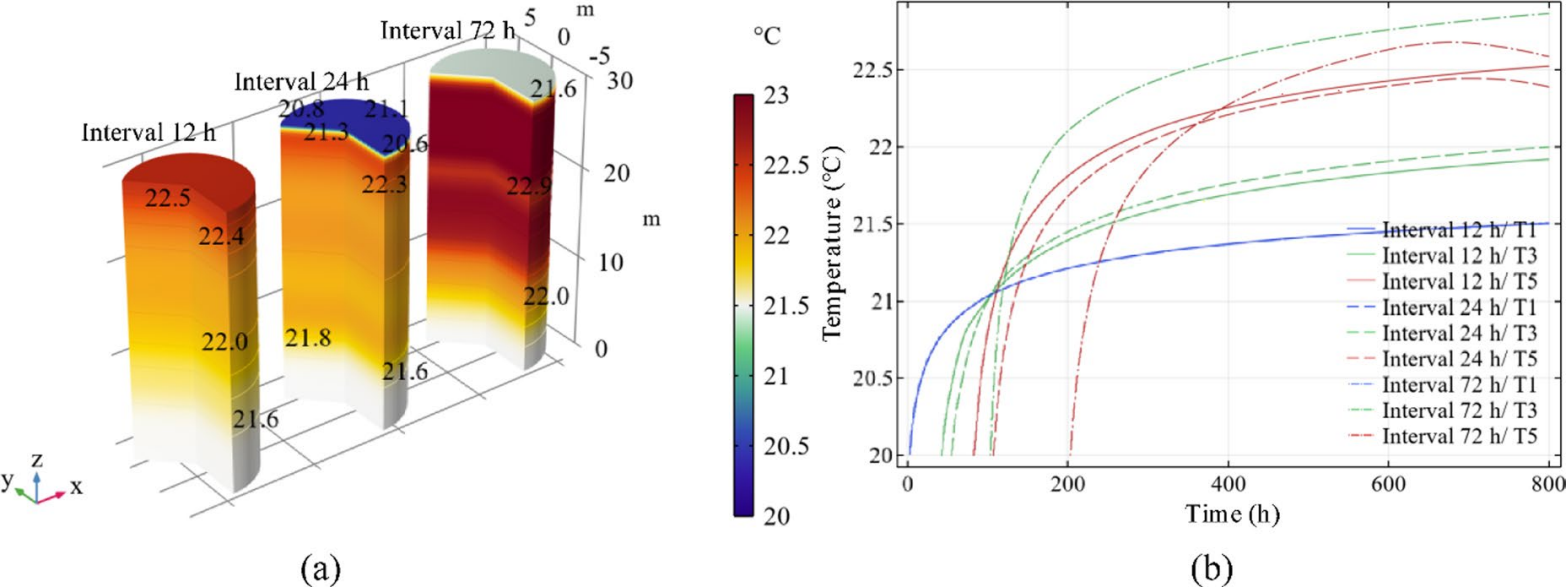
Spatiotemporal evolution of temperature in backfill under different filling interval times. (**a**) Spatial distribution of temperature at 800 h. (**b**) Effect of interval time on temperature.

**Fig. 15:**
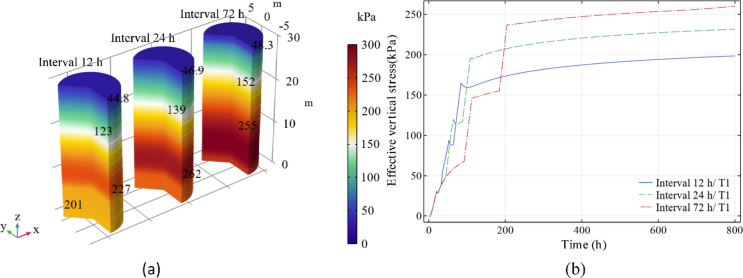
Evolution of effective vertical stress in backfill under different filling interval times. (**a**) Spatial distribution of effective vertical stress at 800 h. (**b**) Effect of interval time on effective vertical stress.

Figures 13, 14 and 15 systematically illustrate the coupled multi-physics influence of different filling interval times (12 h, 24 h, and 72 h) on PWP, temperature field, and effective vertical stress within the CPB. As a critical parameter in the staged filling process, the filling interval time essentially alters the mechanical bearing environment for subsequent layers by modifying the duration of consolidation drainage and hydration reaction of the preceding layer.

First, Fig. 13 reveals the significant regulatory effect of interval time on PWP dissipation. Comparing the spatial distribution in Fig. 13(a) with the monitoring curves in Fig. 13(b), it is evident that extending the filling interval significantly accelerates the PWP dissipation process. Under the long-interval condition of 72 h, the slurry in the preceding layer obtains sufficient resting consolidation time before the next layer is poured, allowing its internal PWP to be fully released (the T1 curve drops the fastest and reaches the maximum negative PWP peak). This provides a low-PWP underlayer boundary for subsequent slurry. Conversely, the short-interval condition of 12 h leads to a significant superposition effect of PWP between new and old slurry layers, causing a marked lag in the dissipation rates at points T1 through T5, and resulting in smaller final negative PWP (matric suction) values. This difference directly determines the effective stress state within the backfill.

Meanwhile, the filling interval time also exerts a non-negligible influence on the temperature field (Fig. 14). Although the final temperatures under various conditions tend to converge, the heating paths exhibit distinct differences. As shown in Fig. 14(b), a long interval time (72 h) leads to a “staggered peak” effect in thermal gradients between new and old backfill layers. Since the preceding layer has partially dissipated heat during the long interval, the subsequent accumulation of heat is relatively gradual. In contrast, under the short-interval (12 h) condition, continuous heat injection makes the thermal accumulation effect within the backfill more concentrated, resulting in a faster early heating rate. Although this difference in thermodynamic behavior is not as drastic as the mechanical response, it indirectly feeds back into the long-term development of strength by affecting the cement hydration rate.

The evolution of effective vertical stress in Fig. 15 is the direct mechanical response to the aforementioned PWP behavior. Following Terzaghi’s effective stress principle, the rapid dissipation of pore water pressure translates directly into the growth of effective stress. As shown in Fig. 15(b), the effective stress at point T1 under the 72 h interval condition increases most rapidly and reaches the highest final value (approaching 250 kPa), significantly outperforming the 12 h and 24 h conditions. This indicates that appropriately extending the interval time facilitates contact and densification between skeleton particles, enabling the bottom of the backfill to form a stable bearing structure more quickly, thereby effectively resisting the risk of failure from newly added upper loads. The contours in Fig. 15(a) also visually demonstrate that as the interval time increases, the high-stress zone (red area) is more widely distributed at the bottom of the backfill, indicating stronger overall mechanical stability. In summary, the 72 h filling interval demonstrates the optimal engineering benefit in promoting PWP dissipation and enhancing effective stress.

## Discussion

### THMC transfer mechanism at layered interfaces

The numerical results indicate that the layered interface is not only a geometric discontinuity generated by the filling sequence, but also a transient zone where hydraulic, thermal, mechanical, and chemical processes interact intensively. During the curing interval, the lower layer experiences hydration, self-desiccation, pore refinement, and stiffness growth. When a new layer is subsequently placed, the lower layer simultaneously acts as a bearing medium, a drainage path, and a thermal storage body. Therefore, the response of the interface controls the redistribution of PWP, the accumulation and dissipation of hydration heat, and the growth of effective stress.

The three-layer strategy shows clear advantages because it divides the total self-weight loading into several stages. Each curing interval provides a drainage and hydration window for the previously placed material. This mechanism explains the sawtooth-like PWP response observed in the simulation. The decrease in PWP during each interval promotes the transformation of total stress into effective stress, thereby improving the early bearing capacity of the CPB. In contrast, continuous filling generates a rapid increase in PWP, and part of the overburden load is temporarily carried by the pore water rather than by the solid skeleton. This leads to a stress-lag effect and a higher risk of barricade loading.

### Comparison with recent studies

Recent experimental studies have shown that the mechanical performance of stratified CPB is significantly affected by the filling interval time, interlayer bonding condition, and the mechanical contrast between adjacent layers^21–25^. For example, studies on non-continuous filling and stratified backfill have reported that an appropriate interval can enhance the strength of the lower layer before the placement of the upper layer, whereas an excessively short interval may lead to insufficient consolidation and weak interlayer performance. The present numerical results are consistent with these findings because the 72 h interval leads to faster PWP dissipation and higher effective vertical stress than the 12 h and 24 h cases.

Compared with recent macro- and meso-scale mechanical studies on layered backfill^23–25^, the present work provides a different but complementary perspective. Previous studies mainly focused on the final strength, failure mode, crack evolution, or uniaxial compression behavior of layered CPB after curing. In contrast, this study focuses on the early-age filling process and reveals how the THMC fields evolve during the “filling-curing-refilling” cycle. Therefore, the present model can explain the formation mechanism of the layered mechanical state before the later-stage failure or strength test.

In addition, the current model improves upon simplified numerical treatments in which layered filling is represented by instantaneous loading or predefined static layers. By introducing the ALE moving mesh method, the rising slurry surface and the time-varying computational domain are explicitly simulated. This is particularly important for capturing the transient peak of PWP and the progressive development of stress during filling. Therefore, the novelty of this study lies in combining a THMC coupling framework with a moving-boundary description of the layered CPB construction process.

### Engineering implications

The simulation results have direct implications for underground mine backfilling design. First, the cement-sand ratio should be selected by balancing strength demand, thermal response, and cost. A high cement-sand ratio can improve early cohesion and promote matric suction development, but it also increases hydration heat and binder consumption. Therefore, it is more suitable for stopes requiring rapid exposure or early self-supporting capacity.

Second, the filling interval time should be designed as a key construction parameter rather than an empirical waiting period. A longer interval can significantly promote PWP dissipation and effective stress development in the lower layer, thereby reducing the risk of barricade failure during subsequent filling. However, an excessively long interval may reduce production efficiency and may generate weak interlayer bonding if the surface becomes too dry or hardened. Therefore, the interval time should be optimized according to stope height, filling rate, binder content, and drainage conditions.

Third, the layering strategy can be used to control the loading path of the backfill. Compared with continuous filling, the three-layer strategy reduces the accumulation of excess PWP and produces a more favorable effective stress distribution at the bottom of the backfill. For high stopes or poor drainage conditions, adopting multi-stage filling can improve operational safety by lowering instantaneous hydraulic pressure and increasing the early bearing capacity of the backfill.

Overall, the proposed model can serve as a numerical tool for optimizing CPB construction parameters. Before field implementation, the model can be calibrated using laboratory and in-situ monitoring data, and then used to predict the evolution of PWP, temperature, and effective stress under different filling schedules. This provides a basis for determining safe filling height, appropriate interval time, and reasonable binder dosage.

## Conclusions and future work

Based on the established THMC multi-field coupling numerical model of CPB, this paper systematically investigates the spatiotemporal evolution laws of multi-physics fields during the layered backfilling process. The main conclusions are as follows:

(1) A multi-field coupling analysis framework for layered backfilling was established. The constructed fully coupled THMC model effectively simulates the “fill-rest” cyclic loading process, accurately capturing the physical field transfer behavior at layered interfaces and the thermo-hydro-mechanical-chemical interaction mechanisms during the transition of the backfill from a fluid state to a solid state.

(2) The controlling role of the c/s ratio on multi-field evolution was elucidated. The c/s ratio dominates the evolution of physical fields by altering the intensity of the hydration reaction. The study found that although a high c/s ratio (1:4) limits the hydration efficiency of single particles due to a low water-cement ratio, the higher cement content per unit volume significantly increases heat generation and water consumption. This results in the highest peak temperature rise (approximately 23 °C) and the rapid formation of matric suction, thereby achieving the highest early cohesion.

(3) The influence law of inter-layer interval time on consolidation characteristics was revealed. The filling interval is a critical window for PWP dissipation. Extending the interval time (from 12 h to 72 h) not only alleviates the heat accumulation effect between new and old slurry layers but, more importantly, allows for the sufficient release of PWP in the underlying backfill. A long interval significantly increases the effective vertical stress at the bottom of the backfill (reaching 250 kPa) by promoting the contact and compaction of skeleton particles, thus enhancing the self-standing stability of the backfill.

(4) The improvement effect of layering strategies on PWP and stress distribution was clarified. The number of layers directly alters the boundary drainage conditions and loading rate of the backfill. Unlike the rapid PWP accumulation (> 100 kPa) caused by continuous filling, the three-layer filling strategy results in a “multi-peak sawtooth-like” dissipation characteristic in PWP evolution, effectively avoiding prolonged periods of high PWP. Simulation results show that the three-layer filling forms the widest high-stress core zone at 800 h, indicating significant advantages in reducing liquefaction risk and improving early bearing capacity.

Although this study has provided insights into the multi-field coupling evolution of backfill, certain limitations regarding model assumptions remain. Future research should be deepened in the following aspects: First, as this study relied on a 2D axisymmetric model focusing on vertical evolution, a 3D THMC coupling model should be established to account for irregular stope cross-sections and 3D spatial effects, thereby more realistically reflecting the influence of complex geometric boundaries. Second, the interaction between the surrounding rock and backfill requires more detailed modeling; subsequent research should move beyond simplified adiabatic and mechanical boundaries to introduce contact mechanics models and hydrothermal exchange mechanisms that consider rock permeability, roughness, and groundwater seepage. Third, to address complex deep mining environments characterized by high ground temperature and stress, chemical damage factors should be incorporated to explore the durability evolution of backfill under long-term service conditions, such as sulfate attack. Finally, relying on actual mining projects, in-situ multi-physics monitoring (temperature, PWP, and stress) should be conducted to further calibrate numerical model parameters using field data, thereby enhancing the model’s applicability in practical engineering.

## Data Availability

The data that support the findings of this study are available from the corresponding author upon reasonable request.

## References

[1] Ansar, F. & Ranjith, P. Innovative green binders: Harnessing mine tailings and by-products for sustainable construction. *J. Build. Eng.* **112**, 113869. 10.1016/J.JOBE.2025.113869 (2025).

[2] Khouia, A. Y. et al. Mitigating contaminated mine drainage through mine waste rock decontamination: A strategy for promoting cleaner and sustainable management. *Miner. Eng.* **225**, 109217. 10.1016/J.MINENG.2025.109217 (2025).

[3] Li, Z. et al. Tailings particle size effects on pollution and ecological remediation: A case study of an iron tailings reservoir. *J. Hazard. Mater.* **476**, 135024. 10.1016/J.JHAZMAT.2024.135024 (2024). 38943882 10.1016/j.jhazmat.2024.135024

[4] Xue, G., Yilmaz, E. & Wang, Y. Progress and prospects of mining with backfill in metal mines in China. *Int. J. Miner. Metall. Mater.* **30**, 1455–1473. 10.1007/S12613-023-2663-0 (2023).

[5] Zhang, P. et al. Use of coal-fired slag in filling bodies with early strength for mining applications. *J. Clean. Prod.*10.1016/J.JCLEPRO.2023.137465 (2023).37101511

[6] Li, S. et al. Current situation and prospects for the clean utilization of gold tailings. *Waste Manag.* **180**, 149–161. 10.1016/J.WASMAN.2024.03.033 (2024). 38569437 10.1016/j.wasman.2024.03.033

[7] Chen, W., Chen, L. & Yin, S. Effect of bacteria-fly-ash based binder on cemented tailings backfill: Mechanical strength, solidified mechanism and economic benefits. *Constr. Build. Mater.* **448**, 138292. 10.1016/J.CONBUILDMAT.2024.138292 (2024).

[8] Hui, C. et al. Research progress and development direction of filling cementing materials for filling mining in iron mines of China. *Gels* **8**(3), 192–192. 10.3390/GELS8030192 (2022). 35323305 10.3390/gels8030192 PMC8954551

[9] Li, C., Li, X. & Ruan, Z. Rheological properties of a multiscale granular system during mixing of cemented paste backfill: A review. *Int. J. Miner. Metall. Mater.* **30**, 1444–1454. 10.1007/s12613-023-2601-1 (2023).

[10] Huizhen, D. et al. Computational fluid dynamics study on cemented paste backfill slurry: Review. *Constr. Build. Mater.*10.1016/J.CONBUILDMAT.2023.130558 (2023).

[11] Yong, W. et al. Experimental research and numerical simulation of the multi-field performance of cemented paste backfill: Review and future perspectives. *Int. J. Miner. Metall. Mater.* **30**(2), 193–208. 10.1007/S12613-022-2537-X (2022).

[12] Cui, L. & Fall, M. A coupled thermo–hydro-mechanical–chemical model for underground cemented tailings backfill. *Tunn. Undergr. Space Technol.* **50**, 396–414. 10.1016/j.tust.2015.08.014 (2015).

[13] Wu, D., Sun, G. & Liu, Y. Modeling the thermo-hydro-chemical behavior of cemented coal gangue-fly ash backfill. *Constr. Build. Mater.* **111**, 522–528. 10.1016/j.conbuildmat.2016.02.179 (2016).

[14] Cui, L. & Fall, M. Multiphysics model for consolidation behavior of cemented paste backfill. *Int. J. Geomech.* **17**(3), 04016077–04016077. 10.1061/(ASCE)GM.1943-5622.0000743 (2016).

[15] Cui Fall. Mathematical modelling of cemented tailings backfill: a review[J].International Journal of Mining, Reclamation and Environment, **33**(6), 389–408. 10.1080/17480930.2018.1453320 (2019).

[16] Cui, L. & Fall, M. An evolutive elasto-plastic model for cemented paste backfill. *Comput. Geotech.* **71**, 19–29. 10.1016/j.compgeo.2015.08.013 (2016).

[17] Wu, D. et al. Numerical analysis of the hydraulic and mechanical behavior of in situ cemented paste backfill. *Geotech. Geol. Eng.* **38**(5), 1–11. 10.1007/s10706-020-01333-2 (2020).

[18] Wenyuan, X. et al. Coupled Effect of Curing Temperature and Moisture on THM Behavior of Cemented Paste Backfill[J]. ADVANCES IN CIVIL ENGINEERING,2020. 10.1155/2020/1870952 (2020).

[19] Wu, D., Fall, M. & Cai, S. Numerical modelling of thermally and hydraulically coupled processes in hydrating cemented tailings backfill columns. *Int. J. Min. Reclam. Environ.* **28**(3), 173–199. 10.1080/17480930.2013.809194 (2014).

[20] Wang, W. et al. Current status of research on fill mining systems. *Recent Pat. Eng.* **19**(8), e240724232252-e240724232252. 10.2174/0118722121305570240722052730 (2025).

[21] Chunming, A. et al. Effect of filling interval time on mechanical characteristics of cemented tailing backfill. *ACS Omega* **8**(46), 43751–43758. 10.1021/ACSOMEGA.3C05652 (2023). 38027356 10.1021/acsomega.3c05652 PMC10666215

[22] Shuaijun, C. et al. Formation mechanism and deformation characteristics of stratified cemented tailings backfill under noncontinuous filling system. *Constr. Build. Mater.*10.1016/J.CONBUILDMAT.2023.131623 (2023).

[23] Zhang, S. et al. Research on the strength influence and crack evolution law of layered backfill based on macro and meso mechanical response. *Constr. Build. Mater.* **449**, 138493–138493. 10.1016/J.CONBUILDMAT.2024.138493 (2024).

[24] Qu, H. et al. Failure behavior and fracture evolution mechanism of layered backfill considering dip angles. *Constr. Build. Mater.* **416**, 135041. 10.1016/J.CONBUILDMAT.2024.135041 (2024).

[25] Xu, W. et al. Mechanical properties, failure modes, and damage development of stratified cemented tailings backfill under uniaxial compression. *Minerals* **14**(9), 917–917. 10.3390/MIN14090917 (2024).

[26] Liu, G. et al. Consolidation process of uncemented backfill slurry in a mine stope considering hydro-geotechnical properties of rockmass in adjacent stopes[J]. **15**(1):24109–24109. 10.1038/S41598-025-08369-5 (Scientific Reports 2025). 10.1038/s41598-025-08369-5 PMC12230130 40619510

[27] Wu, D., Fall, M. & Cai, S. Coupling temperature, cement hydration and rheological behaviour of fresh cemented paste backfill. *Miner. Eng.* **42**, 76–87. 10.1016/j.mineng.2012.11.011 (2013).

[28] Nasir, O. & Fall, M. Coupling binder hydration, temperature and compressive strength development of underground cemented paste backfill at early ages. *Tunn. Undergr. Space Technol. Incorpor. Trenchless Technol. Res.* **25**(1), 9–20. 10.1016/j.tust.2009.07.008 (2009).

[29] Ghirian, A. & Fall, M. Coupled thermo-hydro-mechanical–chemical behaviour of cemented paste backfill in column experiments. Part I: Physical, hydraulic and thermal processes and characteristics. *Eng. Geol.* **164**, 195–207. 10.1016/j.enggeo.2013.01.015 (2013).

[30] Ghirian, A. & Fall, M. Coupled thermo-hydro-mechanical–chemical behaviour of cemented paste backfill in column experiments. *Eng. Geol.* **170**, 11–23. 10.1016/j.enggeo.2013.12.004 (2014).

[31] Chen, Q. et al. Experimental investigation on the strength characteristics of cement paste backfill in a similar stope model and its mechanism. *Constr. Build. Mater.* **154**, 34–43. 10.1016/j.conbuildmat.2017.07.142 (2017).

[32] Somerton, W. H., Keese, J. A. & Chu, S. L. Thermal behavior of unconsolidated oil sands. *SPE J.* **14**, 513–521. 10.2118/4506-PA (1974).

[33] Côté, J. & Konrad, J. A generalized thermal conductivity model for soils and construction materials. *Can. Geotech. J.* **42**(2), 443–458. 10.1139/t04-106 (2005).

[34] Zhang, L., Cai, Z. & Liu, H. A novel approach for simulation of soil-tool interaction based on an arbitrary Lagrangian–Eulerian description. *Soil Tillage Res.* **178**, 41–49. 10.1016/j.still.2017.12.011 (2018).

[35] Xiang, L. W., Hai, S. L. & Chun, F. Lagrange’s equations for seepage flow in porous media with a mixed Lagrangian-Eulerian description. *Acta Mech. Sin.*10.1007/S10409-023-23022-X (2023).

